# Aluminum Toxicity-Induced Alterations of Leaf Proteome in Two Citrus Species Differing in Aluminum Tolerance

**DOI:** 10.3390/ijms17071180

**Published:** 2016-07-21

**Authors:** Huan Li, Lin-Tong Yang, Yi-Ping Qi, Peng Guo, Yi-Bin Lu, Li-Song Chen

**Affiliations:** 1 Institute of Plant Nutritional Physiology and Molecular Biology, College of Resources and Environment, Fujian Agriculture and Forestry University, Fuzhou 350002, China; lihuanenglish123@126.com (H.L.); talstoy@163.com (L.-T.Y.); 6253730@163.com (P.G.); yibin.07@163.com (Y.-B.L.); 2 Fujian Provincial Key Laboratory of Soil Environmental Health and Regulation, College of Resources and Environment, Fujian Agriculture and Forestry University, Fuzhou 350002, China; 3 Institute of Materia Medica, Fujian Academy of Medical Sciences, Fuzhou 350001, China; qiyiping2008@hotmail.com; 4 The Higher Educational Key Laboratory of Fujian Province for Soil Ecosystem Health and Regulation, Fujian Agriculture and Forestry University, Fuzhou 350002, China

**Keywords:** aluminum toxicity, *Citrus grandis*, *Citrus sinensis*, iTRAQ, leaves, proteome

## Abstract

Seedlings of aluminum-tolerant ‘Xuegan’ (*Citrus sinensis*) and Al-intolerant ‘sour pummelo’ (*Citrus grandis*) were fertigated for 18 weeks with nutrient solution containing 0 and 1.2 mM AlCl_3_·6H_2_O. Al toxicity-induced inhibition of photosynthesis and the decrease of total soluble protein only occurred in *C. grandis* leaves, demonstrating that *C. sinensis* had higher Al tolerance than *C. grandis*. Using isobaric tags for relative and absolute quantification (iTRAQ), we obtained more Al toxicity-responsive proteins from *C. sinensis* than from *C. grandis* leaves, which might be responsible for the higher Al tolerance of *C. sinensis*. The following aspects might contribute to the Al tolerance of *C. sinensis*: (a) better maintenance of photosynthesis and energy balance via inducing photosynthesis and energy-related proteins; (b) less increased requirement for the detoxification of reactive oxygen species and other toxic compounds, such as aldehydes, and great improvement of the total ability of detoxification; and (c) upregulation of low-phosphorus-responsive proteins. Al toxicity-responsive proteins related to RNA regulation, protein metabolism, cellular transport and signal transduction might also play key roles in the higher Al tolerance of *C. sinensis*. We present the global picture of Al toxicity-induced alterations of protein profiles in citrus leaves, and identify some new Al toxicity-responsive proteins related to various biological processes. Our results provide some novel clues about plant Al tolerance.

## 1. Introduction

Aluminum is the most abundant metallic element in the Earth’s crust [1]. In neutral or slightly acidic soils, Al is mainly in the form of insoluble deposits and is biologically inactive. In acidic solutions (pH < 5.0), Al exists in the forms of Al^3+^ and Al(OH)^2+^, which are soluble and available to plants [2]. Because micromolar concentration of Al^3+^ can rapidly inhibit root growth, Al toxicity is a major factor limiting crop productivity in many acidic soils through the tropics and subtropics. Over 50% of the world's potential arable lands are acidic [3,4]. Moreover, the acidity of the soils is gradually increasing due to environmental problems, including acid deposition, improper application of chemical fertilizers, intensive agriculture and monoculture [5].

Higher plants have evolved two main mechanisms of Al detoxification (i.e., external and internal detoxification mechanisms) that enable them to tolerate high levels of Al. However, the molecular mechanisms for Al tolerance in higher plants are not fully understood [4,6,7,8,9]. The alteration of proteins is an important process to cope with elevated Al in higher plants [7,10,11,12,13]. Proteome analysis is becoming a powerful tool for the investigation of protein roles in higher plants. There have been several reports investigating Al toxicity-responsive proteins using isobaric tags for relative and absolute quantification (iTRAQ) or two-dimensional gel electrophoresis (2-DE). Wang et al. used iTRAQ to isolate 106 differentially-abundant proteins from Al-treated rice roots [11], indicating that Al toxicity-induced activation of the glycolysis/gluconeogenesis shunt might be a rapid and effective way to balance the available energy levels to prevent the Al toxicity-induced shortage of intracellular energy. Using iTRAQ, Jiang et al. isolated more Al toxicity-responsive proteins from Al-tolerant *Citrus sinensis* roots than from Al-intolerant *Citrus grandis* roots [10], suggesting that the higher metabolic flexibility might contribute to the higher Al tolerance of *C. sinensis*. Zhen et al. used 2-DE to investigate the Al toxicity-induced alterations of root proteome in an Al-resistant soybean cultivar and concluded that proteins related to stress/defense, signal transduction, transport, protein folding, gene regulation and primary metabolism were critical for plant survival under Al toxicity [14]. Yang et al. used 2-DE to identify 12 upregulated and five downregulated proteins from Al toxicity-exposed rice roots. Further analysis showed that cysteine synthase (CS) played a key role in rice Al tolerance [12]. Duressa et al. observed that Al toxicity-induced alterations of root protein profiles differed between Al-tolerant and -sensitive soybean genotypes, concluding that enzymes involved in organic acid biosynthesis and detoxification systems played crucial roles in soybean Al tolerance [15]. Dai et al. isolated 35 proteins related to Al tolerance from Al-tolerant wild barley roots [16]. In tomato roots, proteins involved in detoxification were induced by Al toxicity [17]. Oh et al. showed that Al toxicity increased the abundance of 19 proteins, including *S*-adenosylmethionine synthetase, oxalate oxidase (OXO), malate dehydrogenase (MDH), citrate synthase and ascorbate peroxidase (APX), and decreased the abundance of 28 proteins, including heat shock protein (HSP) 70, *O*-methyltransferase 4 and enolase, in wheat roots [18]. All of these studies, however, have focused on root proteomics, because the inhibition of root growth is one of the earliest and most easily-observed symptoms of Al toxic damage [1]. To our knowledge, data available on the effects of Al toxicity on leaf proteomics are rare [7]. In a study, Rahman et al. used 2-DE to identify eight upregulated proteins, including glutamine synthetase and peroxiredoxin, and nine downregulated ones, including ribulose-1,5-bisphosphate carboxylase/oxygenase (RuBisCO) in high Al-treated leaves [19]. In another study, Yang et al. identified 92 proteins in Al or NO-treated rice leaves. Further analyses confirmed that NO alleviated Al toxicity-induced reactive oxygen species (ROS) and reactive nitrogen species (RNS) toxicities by increasing the activity and abundance of antioxidant enzymes [13].

Citrus is mainly grown in acidic and strong acidic soils. Low pH and high Al are the factors causing the poor growth and decreased lifespan of citrus trees [20]. In 2011, we investigated the pH of 319 soil samples from Pinghe pummelo (*Citrus grandis*) orchards, located in Zhangzhou, China. The pH ranged from 3.26 to 6.22 with an average value of 4.34. Up to 90.0% of the soils displayed a pH of less than 5.0 [21]. During 1998–1999, Huang et al. assayed the pH of 200 soil samples from Pinghe pummelo orchards. The pH ranged from 3.57 to 7.25 with an average value of 4.63. Eighty-five point five percent of the soils had a lower pH than 5.0 [22]. Soil acidification has been occurring rapidly in pummelo orchards in the last decade. Therefore, understanding the mechanisms of Al toxicity and Al tolerance in citrus plants is very important for citrus production. Previously, we investigated the Al toxicity-responsive proteins in the roots of two citrus species differing in Al tolerance [10]. On this basis, we further examined the Al toxicity-induced alterations of gas exchange, Al and total soluble protein concentrations and protein profiles revealed by iTRAQ in Al-tolerant *C. sinensis* and Al-intolerant *C. grandis* leaves [23]. The objectives were (a) to understand the molecular mechanisms of citrus in dealing with Al toxicity at the protein level and (b) to identify proteins conferring Al tolerance in citrus.

## 2. Results

### 2.1. Leaf Gas Exchange, Al and Total Soluble Protein Concentrations

**Figure 1:**
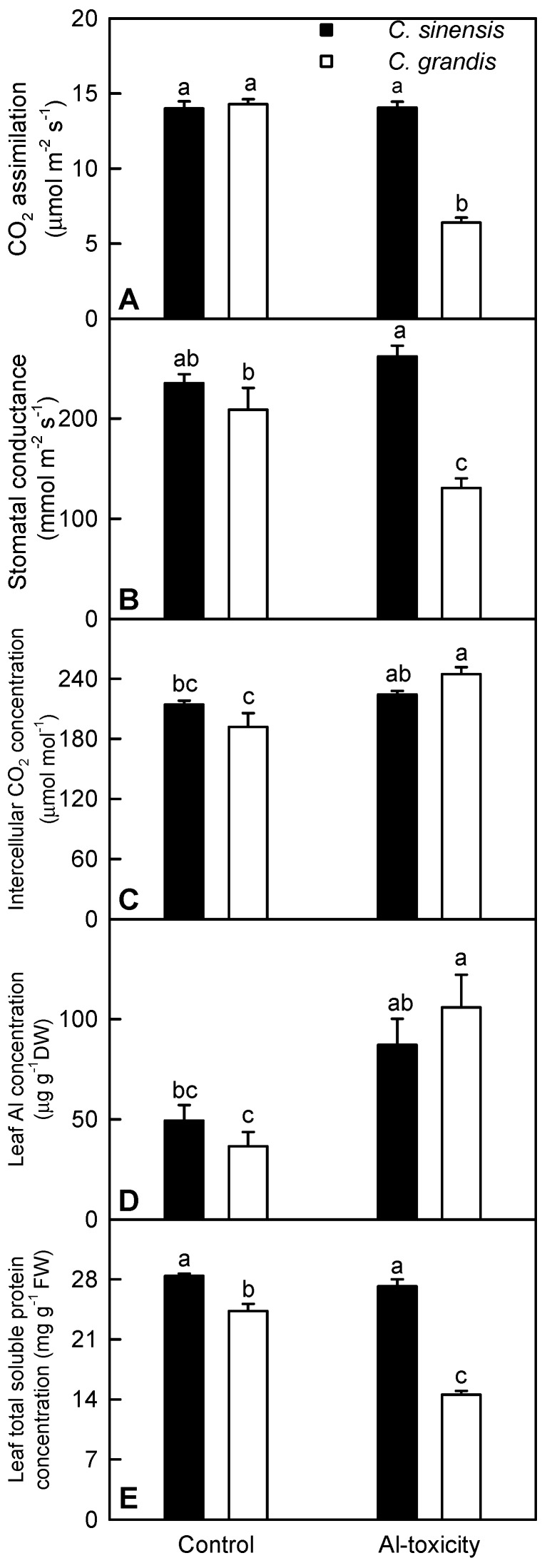
Effects of Al toxicity on leaf CO_2_ assimilation (**A**); stomatal conductance (**B**); intercellular CO_2_ concentration (**C**); Al (**D**) and total soluble protein (**E**) concentrations. Bars represent the means ± standard error SE (*n* = 5). DW: dry weight; FW: fresh weight. Differences among four treatment combinations (two species × two Al) were analyzed by two-way analysis of variance. Means were separated by Duncan’s new multiple range test. Different letters above the bars indicate a significant difference at *p* < 0.05.

We found that +Al *C. grandis* leaves had decreased CO_2_ assimilation and stomatal conductance, but increased intercellular CO_2_ concentration (Figure 1A–C), implying that Al toxicity-induced inhibition of photosynthesis in *C. grandis* was mainly limited by non-stomatal factors. However, Al toxicity had no significant influence on *C. sinensis* leaf gas exchange. Leaf gas exchange parameters did not significantly differ between the two citrus species at each given Al level, except that both CO_2_ assimilation and stomatal conductance were lower in +Al *C. grandis* leaves than in +Al *C. sinensis* leaves (Figure 1A–C). In *C. grandis*, the Al level was higher in +Al leaves than in controls. In *C. sinensis*, the Al level showed an increased trend in +Al leaves, but did not significantly differ between +Al and control leaves. No significant difference was observed in leaf Al concentration between the two citrus species at each given Al treatment (Figure 1D). Besides, Al toxicity only decreased the total soluble protein level in *C. grandis* leaves (Figure 1E). Therefore, *C. sinensis* was more tolerant to Al toxicity than *C. grandis*. A similar result has been obtained by Yang et al. [23] and Jiang et al. [10].

### 2.2. Leaf Al Toxicity-Responsive Proteins

We produced a total of 642,359 and 656,455 spectra from *C. sinensis* and *C. grandis* leaves respectively using iTRAQ (Figures S1A and S2A). Unlike total spectra, the numbers of peptide spectrum matches, peptides identified, unique peptides, proteins identified and protein group(s) identified were higher in *C. sinensis* leaves than in *C. grandis* ones. The protein number in the two citrus species decreased with increased number of peptides that matched to proteins. The number of proteins with the same number of peptides was lower in *C. grandis* leaves than in *C. sinensis* ones (Figures S1B and S2B).

Protein mass distribution, the distribution of sequence coverage and the distribution of peptide length were similar between *C. sinensis* and *C. grandis* leaves. Proteins with 20–30 kDa were the most abundant (Figures S1C and S2C). The protein number in the two citrus species decreased with increased sequence coverage (Figures S2D and S3D). The number of peptides increased as amino acid residues increased from six to eight, was less changed as amino acid residues increased from eight to ten, then decreased with increased amino acid residues (Figures S1E and S2E).

**Figure 2:**
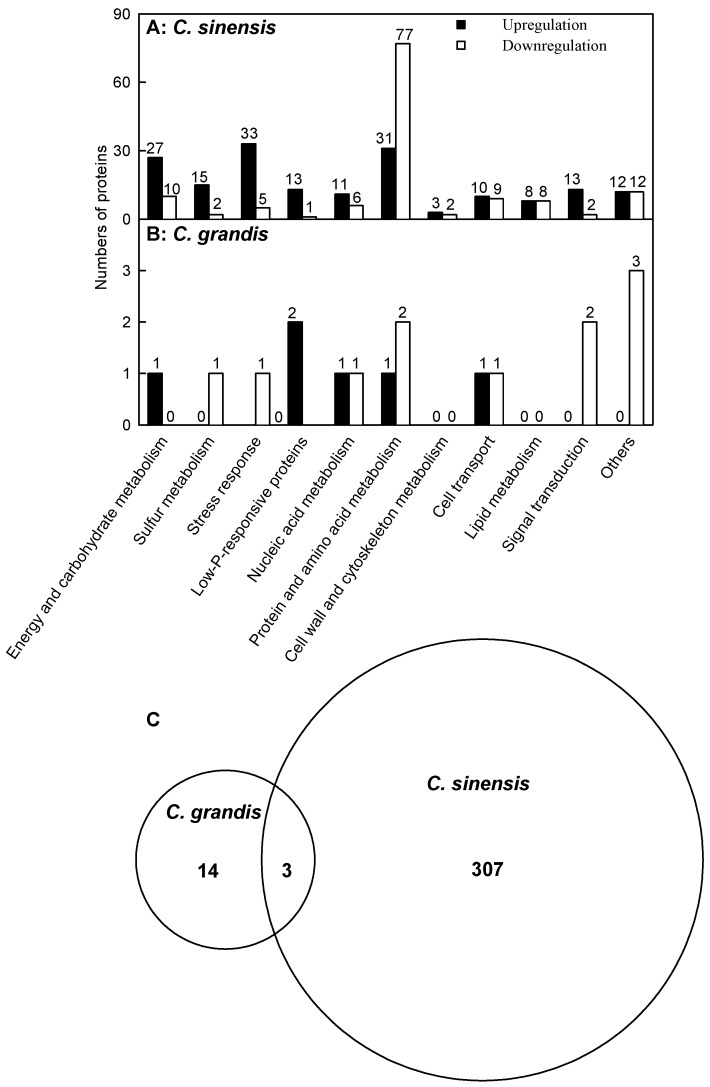
Classification of Al toxicity-responsive proteins in *C. sinensis* (**A**) and *C. grandis* (**B**) leaves; and Venn diagram analysis of Al toxicity-responsive proteins (**C**).

We isolated 176 proteins with increased abundance and 134 proteins with decreased abundance from +Al *C. sinensis* leaves. These proteins were related to energy and carbohydrate metabolism, sulfur metabolism, stress response, low-phosphorus (P) response, nucleic acid metabolism, protein and amino acid metabolism, cell wall and cytoskeleton metabolism, cellular transport, lipid metabolism, signal transduction and other processes. By contrast, we only obtained six up- and 11 downregulated proteins from +Al *C. grandis* leaves. These proteins were associated with energy and carbohydrate metabolism, S metabolism, stress response, low-P response, nucleic acid metabolism, protein and amino acid metabolism, cellular transport, signal transduction and other processes (Figure 2A,B and Tables S1 and S2).

As shown in Figure 2C and Tables S1 and S2, 324 differentially-abundant proteins were found in +Al *C. sinensis* and *C. grandis* leaves. Among these proteins, 307 differentially-abundant proteins only presented in *C. sinensis* and 14 only presented in *C. grandis*, and only three differentially-abundant proteins with the same accession number (i.e., Ciclev10026096 m, Ciclev10015568 m and Ciclev10009194 m) were shared by the two species.

### 2.3. Transcriptional Analysis of Genes for Some Differentially-Abundant Proteins

qRT-PCR was applied to analyze the mRNA levels of genes for 20 differentially-abundant proteins, which were selected in a randomized manner from +Al *C. grandis* and *C. sinensis* leaves. Except for three genes (i.e., Ciclev10008649m, Ciclev10000951m and Ciclev10022212m), the expression profiles of all of these genes matched with our iTRAQ data (Figure S3; Tables S1 and S2), indicating that 85% of these differentially-abundant proteins were regulated at the transcriptional level.

### 2.4. Analysis of Five Al Toxicity-Responsive Enzymes in C. sinensis Leaves

**Figure 3:**
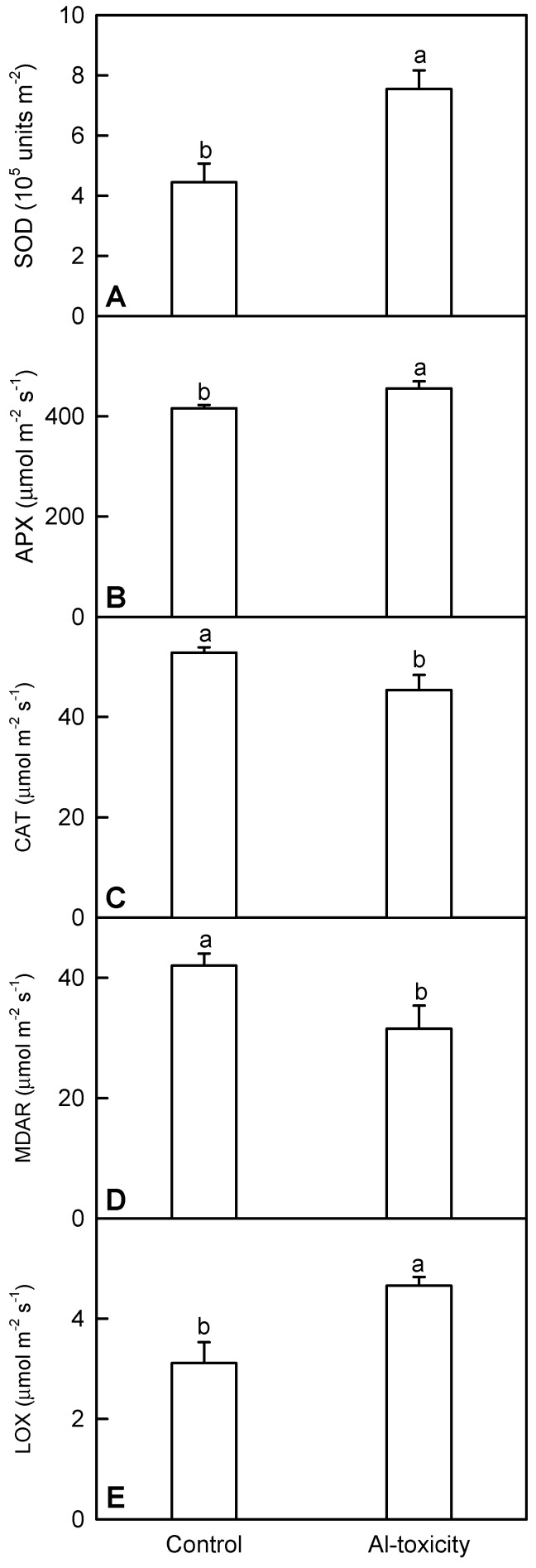
Effects of Al toxicity on the activities of superoxide dismutase (SOD) (**A**); ascorbate peroxidase (APX) (**B**); catalase (CAT) (**C**); monodehydroascorbate reductase (MDAR) (**D**) and lipoxygenase (LOX) (**E**) in *C. sinensis* leaves. Bars represent the means ± SE (*n* = 4). Significance tests for two means (control and Al toxicity) were carried out by the unpaired *t*-test at the *p* < 0.05 level. Different letters above the bars indicate a significant difference at *p* < 0.05.

In this study, we measured the activities of five enzymes related to ROS scavenging (i.e., superoxide dismutase (SOD), APX, catalase (CAT) and monodehydroascorbate reductase (MDAR)) and jasmonic acid (JA) biosynthesis (i.e., lipoxygenase (LOX)) in *C. sinensis* leaves in order to validate the differentially-abundant enzymes. As shown in Figure 3 and Table S1, the activities of all five enzymes matched well with our iTRAQ data.

## 3. Discussion

### 3.1. C. sinensis Displayed Higher Metabolic Flexibility than C. grandis

Alterations of proteome compositions are required for plants to deal with Al [7,10,11,12,13]. As shown in Tables S1 and S2, the number of Al toxicity-responsive proteins was higher in *C. sinensis* leaves than in *C. grandis* leaves, meaning that *C. sinensis* had higher metabolic flexibility than *C. grandis*. This agrees with the report that more Al toxicity-responsive proteins were isolated from *C. sinensis* roots than from *C. grandis* ones [10]. Thus, we concluded that the metabolic flexibility contributed to the higher Al tolerance of *C. sinensis*.

### 3.2. Al Toxicity-Induced Alterations of Energy and Carbohydrate Metabolism-Related Proteins Contribute to the Higher Al-Tolerance of C. sinensis

We isolated 27 up- and 10 downregulated proteins related to energy and carbohydrate metabolism from +Al *C. sinensis* leaves, but only one upregulated proteins from +Al *C. grandis* ones (Tables S1 and S2). A similar result has been obtained on +Al *C. sinensis* and *C. grandis* roots [10]. Energy deprivation is a general symptom of photosynthetic plants under most types of stress. A close relationship exists between energy availability and stress tolerance [24]. Here, we identified eight upregulated proteins in photosystem (PS) II and Calvin cycle from +Al *C. sinensis* leaves, which agrees with the report that Al toxicity increased the activities of Calvin cycle enzymes in citrus leaves [25]. The upregulation of these proteins might be responsible for the higher photosynthesis and for the maintenance of energy homeostasis. This is also supported by our data that CO_2_ assimilation was not reduced in +Al *C. sinensis* leaves (Figure 1A).

Seven increased (i.e., AT5G11720.1, AT3G26720.1, AT4G39010.1, AT4G34480.1, AT3G57240.1, AT3G57270.1 and AT5G24090.1) and two decreased (i.e., AT5G49360.1 and AT3G48950.1) proteins in abundances related to cell wall polysaccharide degradation were isolated from +Al C. *sinensis* leaves. Furthermore, four upregulated proteins in cell wall polysaccharide (i.e., AT1G22360.1 and AT1G22380.1) and starch (i.e., AT2G36390.1 and AT1G32900.1) biosynthesis, one downregulated protein (i.e., AT1G29050.1) in cellulose synthesis and one downregulated protein (i.e., AT5G19220.1) in starch biosynthesis were identified from +Al *C. sinensis* leaves (Table S1). Thus, the remodeling of the cell wall structure might occur in these leaves by selective degradation and/or biosynthesis of some polysaccharide components, thus contributing to their Al tolerance [26]. Besides, the degradation products of the reserve polysaccharides might be utilized as a source of carbon or energy for other key metabolic processes [27].

We found that all four differentially-abundant proteins (i.e., AT3G04120.1, AT5G56350.1, AT3G08590.1 and AT5G15140.1) in glycolysis were upregulated in +Al *C. sinensis* leaves (Table S1). Wang et al. observed that most of the Al toxicity-responsive proteins in glycolysis were upregulated in Al-tolerant rice roots, concluding that Al-tolerant roots could maintain their basic respiration and provide more glycolytically-produced ATP under Al stress [11]. Thus, the Al toxicity-induced upregulation of glycolysis might play a role in the higher Al tolerance of *C. sinensis*.

### 3.3. Al Toxicity-Induced Upregulation of Antioxidant Systems and Other Stress-Related Proteins Played a Role in the Al Tolerance of C. sinensis

Aluminum toxicity can lead to the excessive generation of ROS, thus causing lipid peroxidation in plants [28,29]. Plants have evolved diverse non-enzymatic and enzymatic defense mechanisms to minimize cellular damage caused by ROS. S metabolism is a core pathway for the synthesis of S-containing compounds [7,12]. Through producing different S-containing compounds, such as reduced glutathione (GSH), cysteine, cysteine-rich metal-chelating proteins, glutaredoxins (GRXs) and thioredoxins (TRXs), ATP sulfurylase (ATPS) and other *S*-metabolism-related enzymes play key roles in plant tolerance to abiotic stresses, including Al toxicity [30,31]. Here, we obtained 15 up- and two downregulated proteins associated with S metabolism from +Al *C. sinensis* leaves, indicating that S metabolism was enhanced in these leaves (Table S1; Figure S4). However, we only isolated one downregulated S metabolism-related protein from +Al *C. grandis* leaves (Table S2). This agrees with our report that S metabolism was upregulated in +Al *C. sinensis* and *C. grandis* roots, particularly in the former [10].

Besides S metabolism-related proteins, the abundance of four other antioxidant enzymes was higher in +Al *C. sinensis* leaves (Table S1), which agrees with our report that the activities of antioxidant enzymes were enhanced in +Al citrus leaves [32]. However, the abundances of CAT, ATP1a/ATP1b and probable MDAR were decreased in +Al *C. sinensis* leaves. To validate the reliability of iTRAQ data, we assayed the activities of four differentially-abundant antioxidant enzymes (i.e., SOD, APX, CAT and MDAR) in *C. sinensis* leaves. The activities of SOD and APX were higher in +Al leaves than in controls, while the reverse was the case for the activities of CAT and MDAR (Figure 3A–D). This fully agrees with the data obtained by iTRAQ (Table S1). Furthermore, we isolated 11 other upregulated proteins (i.e., AT4G10720.1, AT5G54620.1, AT2G42590.3, AT3G23400.1, AT4G22240.1, AT3G47860.1, AT1G09560.1, AT4G03240.1 and three AT1G17100.1), which can protect plants against oxidative stress, from +Al *C. sinensis* leaves.

Al toxicity increases the production of aldehydes [29], which can result in a rapid and excessive accumulation of ROS in plant cells [33]. Yin et al. demonstrated the involvement of aldehydes in +Al tobacco roots [29]. Transgenic plants and yeasts overexpressing aldehyde scavenging enzyme genes, such as aldo-keto reductase [34], alcohol dehydrogenase [35] and aldehyde dehydrogenase [33], displayed enhanced tolerance to oxidative stress resulting from various environmental stresses through aldehyde detoxification. We observed that the abundances of one probable aldo-keto reductase 2 and two alcohol dehydrogenases were increased in +Al *C. sinensis* leaves, indicating that aldo-keto reductase and alcohol dehydrogenase might play a role in the adaptation of *C. sinensis* to Al toxicity. However, the abundance of aldehyde dehydrogenase 22A1 was reduced in +Al *C. sinensis* leaves (Table S1).

To sum up, the total ability of detoxification was greatly enhanced in +Al *C. sinensis* leaves. However, only one downregulated S metabolism-related protein was identified from +Al *C. grandis* leaves (Table S2). Therefore, the Al toxicity-induced upregulation of detoxification systems might play a role in the higher Al tolerance of *C. sinensis*.

Chaperones/HSPs play key roles in protecting plants against various stresses, including Al toxicity [7]. We obtained eight upregulated chaperones/HSPs from +Al *C. sinensis* leaves (Table S1), which agrees with the reports that there are one low molecular weight (LMW)-HSP and three DnaJ-like proteins in Al-tolerant soybean roots [14], and two dnaK-type molecular chaperone hsc70.1 (At5g02500) in Al-tolerant *Arabidopsis* ecotype (Col-0) roots increased in response to Al toxicity [36]. Therefore, chaperones/HSPs might play a role in the higher Al tolerance of *C. sinensis* via re-establishing normal protein conformation and maintaining cellular homeostasis. In addition, Al toxicity also induced another seven stress-related proteins (i.e., AT1G01470.1, AT5G54110.1, AT1G17020.1, AT3G04720.1, AT2G21620.1, AT3G53990.1 and AT1G24020.2) in *C. sinensis* leaves.

### 3.4. Low P-Responsive Proteins Were Induced by Al Toxicity, Particularly in C. sinensis Leaves

We isolated one down- and 14 upregulated low P-responsive proteins from +Al *C. sinensis* leaves, but only two upregulated ones from +Al *C. grandis* leaves (Tables S1 and S2). This agrees with our reports that Al toxicity reduced the P level in citrus roots, stem and leaves [23,37]. Inorganic pyrophosphatases, which catalyze the hydrolysis of pyrophosphate (PPi) to phosphate (Pi), may play a role in plant adaptation to Pi- limitation [38]. Ribonucleases have a role in the remobilization of Pi during Pi limitation [39]. Al toxicity leads to plant P deficiency, thus inducing purple acid phosphatases in order to enhance Pi acquisition and utilization in plants [11]. The glycerophosphodiester phosphodiesterase (GDPD)-mediated lipid metabolic pathway might function in the release of Pi from phospholipids during Pi deprivation [40]. Gregory et al. reported that in vivo phosphorylation activation of phosphoenolpyruvate carboxylase contributed to the metabolic adaptation of Pi-starved *Arabidopsis* [41]. Caparrós-Martín et al. found that the expression of *AtSgpp* (At2g38740) encoding haloacid dehalogenase (HAD)-like hydrolase was affected by (a)biotic stresses, being the greatest under Pi starvation, concluding that *AtSgpp* might function in maintaining the homeostatic balance of Pi in the cell [42]. Thus, the Al toxicity-induced upregulation of low P-responsive proteins might play a role in the maintenance of cellular P homeostasis through the conversion of organic P and/or PPi into available Pi.

### 3.5. RNA Regulations Might Play a Role in the Higher Al Tolerance of C. sinensis

Gene expression is regulated at the transcriptional and the post-transcriptional levels. We identified five upregulated transcription factors (TFs) (i.e., probable WRKY TF 50, basic-leucine zipper (bZIP) TF family protein, nuclear factor Y (NFY), subunit C11, C2H2-like zinc finger protein and HAP3-like protein), one downregulated TF (Myb domain protein 15), two upregulated (i.e., methyl-CPG-binding domain 11 and GLNB1 homolog) and two downregulated (i.e., zinc knuckle (CCHC-type) family protein and mitochondrial transcription termination factor family protein) proteins related to transcription regulation in +Al *C. sinensis* leaves (Table S1). Regulatory genes have crucial roles in plant tolerance to abiotic stresses, including Al toxicity [6,7,43,44]. Three zinc finger TFs, *AtSTOP1* (sensitive to proton rhizotoxicity), *OsART1* (Al resistance TF) and *TaSTOP1*, have been cloned from *Arabidopsis*, rice and bread wheat, respectively. They play a key role in Al detoxification via regulating multiple genes responsible for Al tolerance [44,45]. The expression level of bZIP94 TF was higher in Al-tolerant soybean roots than in Al-sensitive ones 48 h after Al treatment [46]. Plant NFY confers maize, rice and *Arabidopsis* stress tolerance [47,48]. The post-transcriptional regulations, which include RNA capping, RNA polyadenylation, RNA splicing, RNA transport and RNA stability, play a role in plant adaptation to abiotic stress, including Al toxicity [49,50]. We got two upregulated RNA-binding (RRM/RBD/RNP motifs) family proteins for mRNA stability, one upregulated pentatricopeptide repeat (PPR) superfamily protein mainly involved in regulating post-transcriptional processes [51], one upregulated putative pre-mRNA splicing factor and one downregulated spliceosome-associated protein 130 for RNA splicing from +Al *C. sinensis* leaves. An *Arabidopsis* chloroplast PPR protein SVR7 was reported to be important for normal photosynthesis and oxidative tolerance [52]. SOAR1, a cytosolic-nuclear PPR protein, had key roles in plant tolerance [51]. Lee et al. showed that STA1, a pre-mRNA splicing factor, was essential for stress tolerance in *Arabidopsis* [53]. To conclude, most of these differentially-abundant proteins related to RNA regulations were induced in +Al *C. sinensis* leaves. However, we only isolated one upregulated methyl-CpG-binding domain 9 and one downregulated DEK domain-containing chromatin-associated protein related to RNA regulations from +Al *C. grandis* leaves (Table S2). Thus, RNA regulation might play a role in the higher Al tolerance of *C. sinensis*.

### 3.6. Protein Metabolism Was More Adaptive to Al Toxicity in C. sinensis than in C. grandis

We isolated 66 down- and nine upregulated proteins in protein synthesis from +Al *C. sinensis* leaves (Table S1), implying that protein synthesis was impaired in these leaves. However, the total soluble protein concentration did not significantly differ between +Al and control *C. sinensis* leaves (Figure 1E). It seemed that other causes were involved in regulating the total soluble protein level in +Al leaves. Cao et al. showed that Al decreased the ATP level in *Pinus massoniana* needles [54]. Thus, the ATP level might be decreased in +Al *C. sinensis* leaves. Protein synthesis, a major consumer of ATP, is subject to strict regulation under conditions where ATP production becomes limited [55]. Thus, the Al toxicity-induced downregulation of protein synthesis-related proteins might be an adaptive response to Al toxicity by saving ATP due to reasonable regulation of protein translation in these leaves. Similar results have been reported on +Al *C. sinensis*, rice and *Arabidopsis* roots [10,11,56]. Furthermore, the Al toxicity-induced inhibition of many ribosomal proteins in *C. sinensis* leaves might imply a redistribution of the resources to meet the increased requirement for amino acids in non-ribosomal peptide (i.e., GSH and phytochelation for Al complexation) synthesis. This is also supported by the above inference that S metabolism was induced in +Al *C. sinensis* leaves and by the report that +Al *Citrus reshni* leaves had higher levels of GSH and oxidized glutathione (GSSG) [32]. However, we only identified two downregulated protein synthesis-related proteins in +Al *C. grandis* leaves (Table S2).

Plant proteases are required for the strict control of protein quality and the selective degradation of specific proteins in response to biotic and abiotic stresses [57]. Futile and inactive (i.e., incorrectly folded) proteins are targeted by ubiquitin for degradation [58]. As expected, we identified 15 upregulated proteinase-related proteins and two upregulated (i.e., AT4G10790.1 and AT4G17510.1) ubiquitination-related proteins in +Al *C. sinensis* leaves (Table S1). In addition to degrading mature proteins into free amino acids, the proteolytic cleavage of proteins by proteinases and ubiquitination also plays a role in the modification and maturation of proteins. Because no difference was observed in the total soluble protein level between +Al and control *C. sinensis* leaves (Figure 1E), the Al toxicity-induced upregulation of proteases and ubiquitination-related proteins might mainly function in the modification and maturation of proteins, which might provide an adaptive response to Al toxicity by maintaining the stability of protein complexes and/or the recycling of nitrogen. By contrast, we only obtained one upregulated ubiquitin-protein ligase 1 from +Al *C. grandis* leaves (Table S2).

### 3.7. Cell Wall and Cytoskeleton Metabolism-Related Proteins

Expansins, which enable the growing cell wall to extend by weakening noncovalent bonding between the matrix and cellulose microfibrils, are believed to be the key regulators of wall extension during growth [59]. The upregulation of expansin-like B1 in +Al *C. sinensis* leaves (Table S1) might be helpful to plant growth by loosening the cell wall. This is supported by our data that the abundance of seven proteins involved in cell wall polysaccharide degradation were enhanced in +Al *C. sinensis* leaves. Al toxicity inhibits cytoskeletal dynamics and Al interacts with the microtubules and actin filaments [60]. As expected, we identified four cytoskeleton-related Al toxicity-responsive proteins from *C. sinensis* leaves. The higher abundance of actin depolymerizing factor 1 in +Al *C. sinensis* leaves means that depolymerization of actin filaments might be enhanced in these leaves. The polymerization and depolymerization of actin filaments may provide cells with the ability to rapidly remodel the cytoskeleton in response to endogenous cues or external signals [61]. Thus, the induction of actin depolymerizing factor 1 in +Al *C. sinensis* leaves might be an adaptive response to Al toxicity.

### 3.8. Cellular Transport-Related Proteins

We identified ten up- and nine downregulated proteins related to cellular transport in +Al *C. sinensis* leaves (Table S1), indicating that cellular transport was altered under Al-stress. Hamilton et al. showed that V-ATPase was induced by Al toxicity in an Al-resistant wheat cultivar [62]. Further study suggested that V-ATPase activity played a role in wheat Al resistance [63]. Ferritins not only are important for iron homeostasis, but also play key roles in preventing oxidative damage by sequestering highly reactive intracellular Fe and inhibiting the production of hydroxyl radicals [64]. Besides their role in the transport of O_2_, plant hemoglobins are plausible targets for enhancing stress tolerance. Over-expression of hemoglobin 1 (Hb1) gene conferred stress tolerance to plants by maintaining the cellular energy status and growth, as well as improving the survival of plants under stress conditions [65,66,67,68]. CDGSH Fe-S domain-containing protein NEET plays crucial roles in plant development, senescence, Fe homeostasis/metabolism and ROS homeostasis [69]. Wang et al. reported that clathrin light chains (CLCs) played a key role in regulating clathrin-mediated trafficking, auxin signaling and development in *Arabidopsis* [70]. Lam et al. demonstrated that the secretory carrier-associated membrane proteins (SCAMPs) highlighted the developing cell plate during cytokinesis in tobacco BY-2 cells [71]. Voltage-dependent anion channels (VDACs) mediate the exchange of metabolites, such as ATP, NADH and ions between mitochondria and cytoplasm. AtVDAC1 is essential for the maintenance of mitochondrial functions associated with energy transaction in *Arabidopsis* [72]. Heavy metal-associated domain-containing protein plays a role in heavy metal transport and/or detoxification [73,74]. Therefore, the Al toxicity-induced upregulation of these proteins might be an adaptive strategy. However, we only isolated one upregulated transport-related protein (AT1G80310.1) from + Al *C. grandis* leaves (Table S2).

### 3.9. Lipid Metabolism-Related Proteins

JA one of the most important signaling molecules, is an oxylipin. Oxylipins and JA derivatives have active roles in plant tolerance to (a)biotic stresses [75]. We found that the levels of three enzymes involved in JA biosynthesis (i.e., lipoxygenase 2 (LOX2), allene oxide cyclase 3 (AOC3) and acyl-activating enzyme 7) were increased in +Al *C. sinensis* leaves, but unaffected in +Al *C. grandis* leaves (Tables S1 and S2). Furthermore, the activity of LOX was increased in +Al *C. sinensis* leaves (Figure 3D). This agrees with the reports that both JA biosynthesis and level might be enhanced in +Al *C. sinensis* roots [10], that both the shoot LOX activity and Al toxicity-induced increase in shoot LOX activity were higher in the Al-tolerant sorghum cultivar than in the Al-sensitive one [76] and that NO enhanced the Al toxicity-induced increase in AOC3 in rice leaves [13]. Therefore, JA signaling might be activated in +Al *C. sinensis* leaves, thus contributing to the higher Al tolerance of *C. sinensis*. Similarly, the levels of two acyl-CoA thioesterases, which presumably participate in the release of JA from JA-CoA [77], were increased in +Al *C. sinensis* leaves. However, the level of long chain acyl-CoA synthetase 4, which activates free fatty acids to acyl-CoA thioesters, was decreased in +Al *C. sinensis* leaves (Table S1). GDSL esterases/lipases perform crucial roles in plant abiotic responses [78]. Gujjar et al. reported that GDSL20 were downregulated more strongly in a drought-sensitive tomato line than in a drought-tolerant one [79]. Thus, the higher level of GDSL esterase/lipase 5 in +Al *C. sinensis* leaves (Table S1) might contribute to the higher Al tolerance of *C. sinensis*.

### 3.10. Signal Transduction-Related Proteins

Signal transduction is altered by Al toxicity [6,7,13]. As expected, we isolated five upregulated and one downregulated protein related to protein phosphorylation/dephosphorylation, four upregulated Ca signal-related proteins and another five proteins (i.e., four upregulated and one downregulated) involved in signal transduction from +Al *C. sinensis* leaves (Table S1). Clay and Nelson found that the loss of *VH1* caused premature leaf senescence and defective vascular transport in *Arabidopsis* [80]. The upregulation of the protein phosphatase 2C (PP2C) family protein in +Al *C. sinensis* leaves agrees with the reports that PP2A was elevated only in Al-tolerant soybean roots by Al toxicity [15] and that the expression of *PP2C* was upregulated in Al-tolerant soybean roots relative to Al-sensitive ones [46]. Studies in transgenic plants demonstrated that serine/threonine-protein phosphatases 2A and 5 positively regulated the responses of plants to abiotic stresses [81,82]. The increased abundance of four Ca signal-related proteins in +Al *C. sinensis* leaves agrees with the report that three Ca-binding proteins were enhanced in +Al rice leaves [13], implying the involvement of Ca in *C. sinensis* Al tolerance. The upregulation of the auxin-responsive family protein in +Al *C. sinensis* leaves indicates that the auxin signal pathway might be involved in plant Al tolerance. This agrees with the report that NO improved rice Al tolerance possibly through its interaction with auxin and GA signals [13]. In addition, we obtained two proteins (i.e., one upregulated farnesylcysteine lyase and one downregulated farnesyltransferase A) involved in abscisic acid (ABA) signaling from +Al *C. sinensis* leaves. To sum up, the responses of *C. sinensis* to Al toxicity were regulated in multiple signal pathways, thus contributing to the higher Al tolerance, while only two downregulated proteins related to signal transduction were isolated from +Al *C. grandis* ones (Table S2).

## 4. Materials and Methods 

### 4.1. Plant Materials and Al Treatments

‘Sour pummelo’ (*Citrus grandis* (L.) Osbeck) seeds were collected from Fujian Academy of Forestry Sciences, Fuzhou, China. ‘Xuegan’ (*Citrus sinensis* (L.) Osbeck) seeds were collected from Minan village, Tingjiang town, Mawei district, Fuzhou, China. This study was carried out at Fujian Agriculture and Forestry University, Fuzhou, China (26°5′ N, 119°14′ E). Plant culture and Al treatments were performed according to Jiang et al. [10]. In late May (five weeks after seed germination), uniform seedlings of *C. grandis* and *C. sinensis* were transported to 6 L pots (two plants to a pot) containing clean river sand and grown in a greenhouse under a natural photoperiod. Each pot was supplied with 500 mL of nutrient solution every two days. The nutrient solution contained the following macronutrients (in mM): KNO_3_, 1; Ca(NO_3_)_2_, 1; KH_2_PO_4_, 0.1; MgSO_4_, 0.5; and micronutrients (in μM): H_3_BO_3_, 20; MnCl_2_, 2; ZnSO_4_, 2; CuSO_4_, 0.5; (NH_4_)_6_Mo_7_O_24_, 0.065; and Fe-ethylenediaminetetraacetic acid (EDTA), 20. Six weeks after transplanting, each pot was supplied daily with a nutrient solution containing 0 (control) or 1.2 mM AlCl_3_·6H_2_O (+Al) until the sand was saturated. The pH of the nutrient solutions was adjusted to 4.1–4.2 using HCl or NaOH. Eighteen weeks after the beginning of Al treatments, fully-expanded (about seven-week-old, midribs and petioles removed) leaves were collected at noon under full sun from different replicates and treatments and immediately frozen in liquid N_2_. Samples were stored at −80 °C until they were used for protein extraction, qRT-PCR analysis, total soluble protein concentration and the enzyme activity assay. The remaining seedlings that were not sampled were used to measure leaf Al concentration and gas exchange.

### 4.2. Measurements of Leaf Gas Exchange, Total Soluble Protein and Al Concentrations

Leaf gas exchange was measured with a CIARS-2 portable photosynthesis system (PP systems, Herts, U.K.) at ambient CO_2_ concentration under a controlled light intensity of ca. 1000 m^−2^·s^−1^ between 9:00 and 11:00 on a clear day. During all of the measurements, leaf temperature and relative humidity were 31.9 ± 0.2 °C and 71.4% ± 0.6%, respectively. There were five replicates per treatment.

Leaf total soluble protein concentration was assayed according to Bradford [83] after being extracted with 50 mM Na_2_HPO_4_-KH_2_PO_4_ (pH 7.0) and 5% (*w*/*v*) insoluble polyvinylpyrrolidone. There were five replicates per treatment.

About seven-week-old leaves (midribs and petioles removed) were collected and dried at 70 °C for 48 h. Leaf Al concentration was determined colorimetrically by the aluminon (the triammonium salt of aurintricarboxylic acid) after being digested in a mixture of HNO_3_:HClO_4_ [84]. There were five replicates per treatment.

### 4.3. Protein Extraction

Proteins were extracted from frozen leaves using a phenol extraction procedure according to Yang et al. [85]. Briefly, equal amounts of frozen leaves from six plants (one plant per pot) were mixed as a biological replicate. There was one biological replicate for each treatment. About 1 g frozen mixed samples was well ground in liquid N_2_ with a mortar and pestle. Four milliliters of ice-cooled buffer containing 100 mM Tris-HCl pH 7.8, 100 mM KCl, 50 mM l-ascorbic acid, 1% (*v*/*v*) Triton X-100, 1% (*v*/*v*) β-mercaptoethanol and 1 mM phenylmethanesulfonyl fluoride (PMSF) were added to the powder and gently pulverized. The mixture was allowed to thaw slowly on ice. The resulting suspension was transferred to a 10-mL tube, then an equal volume of Tris-phenol (pH 8.0) was added. The mixture was thoroughly vortexed before centrifuging at 13,000× *g* for 15 min at 4 °C. The upper phenolic phase was transferred to a 50-mL tube, then five volumes of 100 mM ammonium acetate/methanol were added. After mixing carefully, the mixture was stored at −20 °C overnight. The supernatant was removed carefully after centrifugation at 13,000× *g* for 15 min at 4 °C, then the protein pellets were suspended in 25 mL of ice-cooled methanol for 2 h at −20 °C. Protein pellets were collected by centrifugation at 13,000× *g* for 15 min at 4 °C and then were resuspended in 25 mL of ice-cooled acetone containing 0.1% β-mercaptoethanol and kept at −20 °C for 2 h. After centrifugation at 13,000× *g* for 15 min at 4 °C, the pellets were washed twice with 25 mL of ice-cooled acetone, then dried by lyophilization and finally stored at −80 °C until use. Lyophilized proteins were dissolved in buffer containing 8 M urea, 50 mM triethylammonium bicarbonate (TEAB), pH 8.5, 0.1% sodium dodecyl sulfate (SDS) and protease inhibitor cocktail (Roche, Indianapolis, IN, USA) for 1 h at 4 °C under constant mixing. After being centrifuged at 16,000× *g* for 20 min at 4 °C, the supernatant was collected and quantified using the BCA assay kit (Pierce, Rockford, IL, USA).

### 4.4. iTRAQ Analysis

iTRAQ analysis was performed according to the manufacturer’s instructions (AB Sciex Inc., Framingham, MA, USA) at CapitalBio Technology, Beijing. Six volumes of pre-cooled acetone (−20 °C) were added to each sample tube (100 μg total protein). After being inverted thrice, the tube was incubated at −20 °C until a flocculent was formed (4–16 h). Thereafter, the acetone was decanted after centrifugation at 10,000× *g* for 15 min. Protein from +Al and control leaves was dissolved in a mixture (100 μL) containing 35 μL 8 M urea and 9 μL 500 mM TEAB. Protein reduction, cysteine block, trypsin digestion and iTRAQ™ labels were performed according to manufacturer’s protocol for iTRAQ^®^ Reagents–8plex (AB Sciex Inc., MA, USA; Sciex iChemistry^®^ Product Number 4390812). Al-treated and control samples for *C. sinensis* were labeled with 121 and 119 tags; samples of *C. grandis* were labeled with 117 and 114 tags, respectively. Each label contained a reporter group, a peptide reactive group (PRG) and a balance group. When the labeled peptide was fragmented along the peptide backbone by MS/MS fragmentation, the iTRAQ™ reporter groups broke off and yielded distinct ions at *m*/*z* 113, 114, 115, 116, 117, 118, 119 and 121. The relative intensities of the reporter ions were shown to be directly proportional to the relative levels of each peptide in the samples. The peptides labeled with the isobaric tags were incubated at room temperature for 2 h. The labeled peptide mixtures were then pooled and stored at −80 °C until use.

For strong cationic exchange (SCX) chromatography using an Agilent 1260 Infinity high-performance liquid chromatography (HPLC) (Agilent Technologies, Palo Alto, CA, USA), the labeled peptides were first lyophilized and reconstituted in solvent A (2% acetonitrile (ACN), pH 10), then the samples were loaded onto the XBridge C18, 5 μm, 250 × 4.6 mm column (Waters, Milford, MA, USA) and eluted using a gradient of 5%–45% Solvent B (90% ACN, pH 10) for 40 min. A total of 40 fractions was collected, which were then concatenated to 20 fractions, vacuum dried and stored at −80 °C until further LC-MS analysis.

The LC-MS/MS analysis was carried out in CapitalBio Technology using a Q Exactive mass spectrometer (Thermo Scientific, San Jose, CA, USA). The peptide mixture was separated by reversed phase chromatography on a DIONEX nano-UPLC system using an Acclaim C18 PepMap100 nano-Trap column (75 μm × 2 cm) connected to an Acclaim PepMap RSLC C18 analytical column (75 μm × 25 cm, 2 μm particle size) (Thermo Scientific). Before loading, the sample was dissolved in Mobile Phase A, containing 2% ACN and 0.1% formic acid. A linear gradient of Mobile Phase B (0.1% formic acid in 99.9% ACN) from 2%–35% in 45 min was followed by a steep increase to 80% Mobile Phase B in 1 min at a flow rate of 300 nL·min^−1^. The nano-LC was coupled online with the Q Exactive mass spectrometer using a stainless steel Emitter coupled to a nanospray ion source.

Mass spectrometry analysis was made in a data-dependent manner with full scans (350–1600 *m*/*z*) acquired using an Orbitrap mass analyzer (Thermo Fisher Scientific, Carlsbad, CA, USA) at a mass resolution of 70,000 FWHM at 400 *m*/*z* in Q Exactive. The twenty most intense precursor ions from a survey scan were selected for MS/MS from each duty cycle and detected at a mass resolution of 17,500 FWHM at *m*/*z* of 400 in the Orbitrap analyzer. All of the tandem mass spectra were produced by the higher energy collision dissociation (HCD) method. Dynamic exclusion was set for 20 s.

Proteome discoverer (1.4) software (Thermo Scientific) was used to perform database searching against the *Citrus clementina* database (https://www.citrusgenomedb.org/species/clementina/genome1.0) using the Sequest algorithms. The following settings were applied: precursor mass tolerance of 20 ppm, fragment mass tolerance of 0.02 Da. Trypsin was specified as the digesting enzyme, and 2 missed cleavages were allowed. Cysteine carbamidomethylation and iTRAQ modifications (N-terminus and lysine residues) were defined as fixed modifications, and methionine oxidation was the variable modification. The results were filtered using the following settings: only high confident peptides with a global false discovery rate (FDR) <1% based on a target-decoy approach were included in the results. In the iTRAQ quantitation workflow, the most confident centroid method was used with an integration window of 20 ppm. For protein quantitation, only proteins that contained at least two unique peptides were used to quantify proteins. The quantitative protein ratios were weighted and normalized by the median ratio in Mascot [10,86]. In this study, a protein was considered differentially abundant when it had a fold change of >2 and a *p* value of <0.05.

Bioinformatic analysis of proteins was performed according to Yang et al. [85] and Gan et al. [87].

### 4.5. qRT-PCR Analysis of Gene Expression

About 300 mg of frozen leaves collected equally from six pots (one plant per pot, one leaf per plant) were mixed as a biological replicate. There were three biological replicates for each treatment. Total RNAs were independently extracted thrice from +Al and control frozen leaves using the Recalcitrant Plant Total RNA Extraction Kit (Centrifugal column type, Bioteke Corporation, Beijing, China) according to the manufacturer’s instructions. Gene-specific primers were designed using Primer Software Version 5.0 (PREMIER Biosoft International, Palo Alto, CA, USA) according to the corresponding sequences of selected proteins in the citrus genome (http://www.phytozome.net/cgi-bin/gbrowse/citrus/). The sequences of the F and R primers used are given in Table S3. qRT-PCR was performed according to Zhou et al. [88]. Each sample was run in two technical replicates. For the normalization of gene expression, citrus *actin* (GU911361.1) was used as an internal standard, and the leaves from control plants were used as the reference sample, which was set to 1.

### 4.6. Analysis of SOD, APX, CAT, MDAR and LOX Activities in C. sinensis Leaves

Leaf SOD, APX, CAT and MDAR were assayed according to Li et al. [89]. Leaf LOX was assayed by the formation of conjugated dienes from linoleic acid according to Axelrod et al. [90].

### 4.7. Experimental Design and Statistical Analysis

There were 40 seedlings (20 pots) in a completely randomized design. Experiments were performed with 3–5 replicates except for iTRAQ analysis. For each treatment, only one biological sample was used to perform iTRAQ analysis. The replicates represented material from individual plants, except for iTRAQ and qRT-PCR analysis, in which each biological replicate was created by pooling equal samples from six different plants (one plant per pot). Differences among four treatment combinations (two species × two Al) were analyzed by two-way analysis of variance. Four means were separated by Duncan’s new multiple range test at *p* < 0.05. Significant tests between two means (control and Al toxicity) were carried out by the unpaired *t*-test at the *p* < 0.05 level.

### 4.8. Data Deposit

The mass spectrometry proteomics data have been deposited to the ProteomeXchange Consortium via the PRIDE Proteomics Identification (PRIDE) partner repository with the dataset identifier PXD002916

## 5. Conclusions

Al toxicity only lowered *C. grandis* leaf CO_2_ assimilation and total soluble protein concentration, demonstrating that *C. sinensis* had higher Al tolerance than *C. grandis.* Here, we used iTRAQ to investigate comparatively Al toxicity-responsive proteins in Al-tolerant *C. sinensis* and Al-intolerant *C. grandis* leaves and obtained more differentially-abundant proteins from +Al *C. sinensis* leaves than from +Al *C. grandis* leaves. The majority of the differentially-abundant proteins only presented in *C. sinensis* or *C. grandis*; only three Al toxicity-responsive proteins were shared by both. *C. sinensis* displayed higher metabolic flexibility than *C. grandis*, possibly contributing to the higher Al tolerance of *C. sinensis*. The higher Al tolerance of *C. sinensis* might include several aspects: (a) photosynthesis and energy-related proteins were more adaptive to Al toxicity in *C. sinensis* than in *C. grandis*, which might account for the better maintenance of photosynthesis and energy balance in +Al *C. sinensis* leaves; (b) less increased requirement for detoxification of ROS and other toxic compounds, such as aldehydes, due to Al toxicity-induced inhibition of photosynthesis, because CO_2_ assimilation was not significantly altered, and great improvement of the total ability of detoxification via inducing proteins related to detoxification of ROS (i.e., S metabolism related proteins, SOD, peroxidase, plastid-lipid-associated proteins, germin-like proteins and frataxin) and aldehydes (i.e., aldo-keto reductase and alcohol dehydrogenase) in +Al *C. sinensis* leaves; (c) induction of low P-responsive proteins in +Al *C. sinensis* leaves. In addition, Al toxicity-responsive proteins related to RNA regulations, protein metabolism, cellular transport and signal transduction might contribute to the higher Al tolerance of *C. sinensis*. Here, we presented the global picture of Al toxicity-induced protein alterations in Al-tolerant *C. sinensis* and Al-intolerant *C. grandis* leaves and identified some new Al toxicity-responsive proteins involved in carbohydrate and energy metabolism (i.e., ferredoxin 3 and aldose 1-epimerase), detoxification (i.e., ATPS, lipocalin, aldo-keto reductase, ankyrin repeat-containing protein and frataxin), low P-response (i.e., ribonuclease and purple acid phosphatase), nucleic acid metabolism (i.e., bZIP TF and HAP3-like protein), protein and amino acid metabolism (i.e., cystatin, arginase and cysteine proteinases), cellular transport (i.e., ferritin and CDGSH iron-sulfur domain-containing protein NEET) and signal transduction (i.e., VH1-interacting kinase and farnesylcysteine lyase) from plant leaves. Thus, our findings will increase our understanding of the molecular mechanisms on citrus Al toxicity and Al tolerance at the protein level.

## Supplementary Materials

Supplementary materials can be found at http://www.mdpi.com/1422-0067/17/7/1180/s1.
