# Supplementary material for: Aluminum Toxicity-Induced Alterations of Leaf Proteome in Two Citrus Species Differing in Aluminum Tolerance
Source: Int J Mol Sci. 2016 Jul 21;17(7):1180. doi: 10.3390/ijms17071180 (PMC4964550; doi:10.3390/ijms17071180)
Supplement: Supplementary file 1 [file ijms-17-01180-s001.pdf]

Supplementary Materials: Aluminum Toxicity-Induced Alterations of Leaf Proteome in Two Citrus Species Differing in Aluminum Tolerance

Huan Li, Lin-Tong Yang, Yi-Ping Qi, Peng Guo, Yi-Bin Lu and Li-Song Chen

Table S1. List of differentially expressed proteins in Al-toxic *Citrus sinensis* leaves. Control and Al-toxic samples were labeled with 119 and 121 tags, respectively.

Accession	Homology in *Arabidopsis thaliana*	Proteins	Fold Change (121/119)
**Energy and carbohydrate metabolism**			
Ciclev10016525m|PACid:20819165	AT4G05180.1	Photosystem II subunit Q-2	2.138
Ciclev10017247m|PACid:20815638	AT3G21055.1	Photosystem II subunit T	2.328
Ciclev10005974m|PACid:20791469	AT3G22840.1	Chlorophyll A-B binding family protein	2.243
Ciclev10002532m|PACid:20787444	AT1G54500.1	Rubredoxin family protein	122.070
Ciclev10017081m|PACid:20817328	AT2G27510.1	Ferredoxin 3	2.190
Ciclev10029499m|PACid:20813098	AT2G27510.1	Ferredoxin 3	2.689
Ciclev10031620m|PACid:20804829	AT2G39730.2	Ribulose bisphosphate carboxylase/oxygenase activase, chloroplastic	370.071
Ciclev10005029m|PACid:20791411	AT1G79530.1	Glyceraldehyde-3-phosphate dehydrogenase	2.635
Ciclev10014974m|PACid:20817138	AT3G07670.1	Rubisco methyltransferase family protein	0.451
Ciclev10014196m|PACid:20818257	AT5G11720.1	α-glucosidase//Glycosyl hydrolases family 31 protein	2.086
Ciclev10017122m|PACid:20815005	AT3G26720.1	Glycosyl hydrolase family 38 protein	2.203
Ciclev10028301m|PACid:20813445	AT4G39010.1	Endo-1,4-β-glucanase//Endoglucanase 24	121.841
Ciclev10028656m|PACid:20812272	AT4G34480.1	(1->3)-β-glucan endohydrolase 7//β-1,3-endoglucanase 7//Glucan endo-1,3-β-glucosidase 7//O-Glycosyl hydrolases family 17 protein	2.260
Ciclev10005195m|PACid:20790202	AT3G57240.1	(1->3)-β-glucan endohydrolase//β-1,3-endoglucanase	2.439
Ciclev10005316m|PACid:20792235	AT3G57270.1	(1->3)-β-glucan endohydrolase//β-1,3-endoglucanase	2.708
Ciclev10005491m|PACid:20790391	AT5G24090.1	Acidic endochitinase	3.017
Ciclev10004369m|PACid:20791173	AT5G49360.1	Arabinofuranosidase//β-D-xylosidase 1	0.388
Ciclev10004864m|PACid:20791543	AT3G48950.1	Pectin lyase-like superfamily protein	0.439
Ciclev10031404m|PACid:20803253	AT1G22360.1	UDP-glucosyl transferase 85A2	171.963
Ciclev10031408m|PACid:20802707	AT1G22380.1	UDP-glucosyl transferase 85A3	40.098
Ciclev10000272m|PACid:20786660	AT2G36390.1	1,4-α-glucan-branching enzyme//Q-enzyme	2.457
Ciclev10019346m|PACid:20806605	AT1G32900.1	GBSSII-2//Granule-bound starch synthase 1, chloroplastic/amyloplastic//Granule-bound starch synthase I	2.211
Ciclev10015687m|PACid:20818911	AT1G29050.1	Trichome birefringence-like 38	0.420
Ciclev10004731m|PACid:20790470	AT5G19220.1	ADP glucose pyrophosphorylase large subunit 1	0.467
Ciclev10032014m|PACid:20805824	AT3G04120.1	Glyceraldehyde-3-phosphate dehydrogenase C subunit 1	2.262
Ciclev10019997m|PACid:20811450	AT5G56350.1	Pyruvate kinase family protein	2.481
Ciclev10011402m|PACid:20797055	AT3G08590.1	Phosphoglycerate mutase, 2,3-bisphosphoglycerate-independent	2.354
Ciclev10011989m|PACid:20798577	AT5G15140.1	Aldose 1-epimerase	16.397

Table S1. *Cont.*

Accession	Homology in *Arabidopsis thaliana*	Proteins	Fold Change (121/119)
**Energy and carbohydrate metabolism**			
Ciclev10022953m|PACid:20808910	AT3G62810.1	Complex 1 family protein/LVR family protein	12.567
Ciclev10015546m|PACid:20818719	AT4G22260.1	Alternative oxidase family protein	2.043
Ciclev10021931m|PACid:20811193	AT4G19540.1	IND1(iron-sulfur protein required for NADH dehydrogenase)-like	2.474
Ciclev10021162m|PACid:20807552	AT5G24400.1	NagB/RpiA/CoA transferase-like superfamily protein	2.126
Ciclev10009694m|PACid:20793497	AT1G18270.3	Ketose-bisphosphate aldolase class-II family protein	0.459
Ciclev10010343m|PACid:20793499	AT5G20830.2	Nodulin-100//Sucrose synthase	0.298
Ciclev10020692m|PACid:20809157	AT3G14420.2	Aldolase-type TIM barrel family protein	0.495
Ciclev10018748m|PACid:20811584	AT1G78580.1	Trehalose-6-phosphate synthase	0.494
Ciclev10016324m|PACid:20816626	AT1G28960.1	Coenzyme A diphosphatase NUDT15//Nudix hydrolase 15, mitochondrial//Predicted protein//nudix hydrolase homolog 15	0.445
**S metabolism**			
Ciclev10004927m|PACid:20791536	AT3G22890.1	ATP sulfurylase 1	3.166
Ciclev10032031m|PACid:20803628	AT3G03630.1	Cysteine synthase 26	9.028
Ciclev10032680m|PACid:20805511	AT1G10370.1	Glutathione *S*-transferase family protein	1682.737
Ciclev10012631m|PACid:20798866	AT5G02790.1	Glutathione *S*-transferase family protein	3.157
Ciclev10022212m|PACid:20809459	AT2G47730.1	Glutathione *S*-transferase F8, chloroplastic	3.548
Ciclev10030408m|PACid:20812962	AT2G29420.1	Glutathione *S*-transferase tau 7	5.701
Ciclev10030418m|PACid:20812954	AT2G29420.1	Glutathione *S*-transferase tau 7	5.328
Ciclev10022164m|PACid:20810119	AT1G78380.1	Glutathione *S*-transferase TAU 19	2.056
Ciclev10009304m|PACid:20794954	AT4G31870.1	Glutathione peroxidase 7	2.220
Ciclev10022130m|PACid:20808338	AT4G11600.1	Glutathione peroxidase 6	2.542
Ciclev10000728m|PACid:20785479	AT5G27380.1	Glutathione synthetase 2	384.080
Ciclev10008149m|PACid:20793255	AT4G33030.1	UDP-sulfoquinovose synthase, chloroplastic	2.061
Ciclev10016894m|PACid:20815716	AT3G54900.1	Monothiol glutaredoxin-S14, chloroplastic	22.681
Ciclev10002859m|PACid:20787371	AT3G51030.1	Thioredoxin H-type 1	2.264
Ciclev10002332m|PACid:20785936	AT1G76020.1	Thioredoxin superfamily protein	2.015
Ciclev10007750m|PACid:20792997	AT4G29210.1	γ-glutamyl transpeptidase 4	0.479
Ciclev10017254m|PACid:20817743	AT1G31170.4	Sulfiredoxin	0.405
**Stress response**			
Ciclev10001726m|PACid:20787677	AT1G71695.1	ATP4a//PRXR6//Peroxidase 12	3.198
Ciclev10012554m|PACid:20797449	AT3G09640.1	Ascorbate peroxidase	2.785
Ciclev10002765m|PACid:20789304	AT1G08830.1	Superoxide dismutase [Cu-Zn]//copper/zinc superoxide dismutase 1	2.641
Ciclev10032661m|PACid:20804250	AT3G10920.1	Mn/Fe superoxide dismutase//Superoxide dismutase [Mn], mitochondrial	2.518
Ciclev10000951m|PACid:20786358	AT4G35090.1	Catalase	0.481
Ciclev10015870m|PACid:20817415	AT4G21960.1	ATP1a/ATP1b//PRXR1	0.387
Ciclev10015090m|PACid:20818685	AT3G27820.1	Probable monodehydroascorbate reductase, cytoplasmic isoform 2	0.301
Ciclev10020289m|PACid:20810123	AT4G10720.1	Ankyrin repeat family protein//Ankyrin repeat-containing protein At2g01680	2.614
Ciclev10024221m|PACid:20810180	AT5G54620.1	Ankyrin repeat family protein//Ankyrin repeat protein//Ankyrin repeat-containing protein At2g01680	2.159
Ciclev10016314m|PACid:20818806	AT2G42590.3	14-3-3 protein homolog [*Maackia amurensis*]//14-3-3-like protein D//SGF14D//General regulatory factor 9	738.459

Table S1. *Cont.*

Accession	Homology in *Arabidopsis thaliana*	Proteins	Fold Change (121/119)
**Stress response**			
Ciclev10007095m|PACid:20790772	AT3G23400.1	Probable plastid-lipid-associated protein 6, chloroplastic	3.571
Ciclev10015934m|PACid:20819295	AT4G22240.1	Probable plastid-lipid-associated protein 2, chloroplastic	2.171
Ciclev10008835m|PACid:20793494	AT3G47860.1	Apolipoprotein D//Chloroplastic lipocalin	2.249
Ciclev10029251m|PACid:20813153	AT1G09560.1	Germin-like protein 5	3.130
Ciclev10016799m|PACid:20818836	AT4G03240.1	Frataxin, mitochondrial	20.281
Ciclev10022174m|PACid:20810231	AT1G17100.1	SOUL heme-binding family protein	2.802
Ciclev10024466m|PACid:20811465	AT1G17100.1	SOUL heme-binding family protein	2.676
Ciclev10022064m|PACid:20808330	AT1G17100.1	SOUL heme-binding family protein	2.251
Ciclev10029029m|PACid:20813663	AT1G60680.1	NAD(P)-linked oxidoreductase superfamily protein//Probable aldo-keto reductase 2	18.580
Ciclev10015901m|PACid:20815543	AT5G19440.1	Alcohol dehydrogenase-like protein	20.223
Ciclev10023259m|PACid:20809337	AT3G03080.1	Alcohol dehydrogenase, putative//NADP-dependent alkenal double bond reductase P2	3959.958
Ciclev10004594m|PACid:20790507	AT3G66658.2	Aldehyde dehydrogenase 22A1	0.405
Ciclev10019095m|PACid:20809601	AT5G52640.1	Heat shock protein 83//Heat shock protein 90.1	2.511
Ciclev10029467m|PACid:20813389	AT3G46230.1	17.4 kDa class I heat shock protein	2.472
Ciclev10007067m|PACid:20790805	AT4G15802.1	Heat shock factor binding protein	543.235
Ciclev10025114m|PACid:20801969	AT1G16030.1	Heat shock 70 kDa protein 5//Heat shock protein 70-5//Heat shock protein, putative	2.146
Ciclev10026390m|PACid:20801494	AT1G52560.1	26.5 kDa heat shock protein, mitochondrial//HSP20-like chaperones superfamily protein//Small heat-shock protein, putative	2.994
Ciclev10024164m|PACid:20808067	AT1G53280.1	Chaperone protein YajL//Class I glutamine amidotransferase-like superfamily protein	508.524
Ciclev10021210m|PACid:20811903	AT1G53280.1	Chaperone protein YajL//Class I glutamine amidotransferase-like superfamily protein	18.328
Ciclev10029386m|PACid:20812277	AT2G16600.1	Peptidyl-prolyl *cis-trans* isomerase CYP19-1	2.267
Ciclev10010508m|PACid:20794622	AT1G01470.1	Late embryogenesis abundant protein	222.637
Ciclev10009203m|PACid:20796146	AT5G54110.1	Membrane-associated mannitol-induced	92.662
Ciclev10012028m|PACid:20798669	AT1G17020.1	Protein SRG1//Senescence-related gene 1	9.830
Ciclev10029528m|PACid:20813912	AT3G04720.1	Pathogenesis-related 4	3.179
Ciclev10029375m|PACid:20814005	AT2G21620.1	Adenine nucleotide α hydrolases-like superfamily protein	2.092
Ciclev10029414m|PACid:20814816	AT3G53990.1	Adenine nucleotide α hydrolases-like superfamily protein//Universal stress protein 1//Universal stress protein A-like protein	2.567
Ciclev10006096m|PACid:20791940	AT1G24020.2	MLP-like protein 423//Major allergen Pru ar 1//Stress-related protein	2.279
Ciclev10032973m|PACid:20802498	AT1G24020.1	MLP-like protein 423//Major latex-like protein	0.407
**Low-P-responsive proteins**			
Ciclev10026096m|PACid:20800141	AT1G73010.1	Inorganic pyrophosphatase 1//Predicted protein	26.117
Ciclev10012723m|PACid:20796310	AT3G53620.1	Soluble inorganic pyrophosphatase 4	2.770
Ciclev10016242m|PACid:20815326	AT2G39780.2	Ribonuclease 2	3.253
Ciclev10016283m|PACid:20815386	AT1G25230.1	Purple acid phosphatase 4	2.776
Ciclev10015905m|PACid:20817929	AT2G01890.1	Purple acid phosphatase 8	2.149
Ciclev10013402m|PACid:20797645	AT3G52780.1	Purple acid phosphatases superfamily protein	2.821
Ciclev10030896m|PACid:20803277	AT1G13900.1	Purple acid phosphatases superfamily protein	2.346
Ciclev10015568m|PACid:20815289	AT3G02040.1	Glycerophosphodiester phosphodiesterase GDE1	7.013
Ciclev10020540m|PACid:20807368	AT5G08030.1	Glycerophosphoryl diester phosphodiesterase//PLC-like phosphodiesterases superfamily protein	2.366

Table S1. *Cont.*

Accession	Homology in *Arabidopsis thaliana*	Proteins	Fold Change (121/119)
**Low-P-responsive proteins**			
Ciclev10018719m|PACid:20805954	AT1G53310.3	Phospho*enol*pyruvate carboxylase 3	2.706
Ciclev10012732m|PACid:20798969	AT1G79790.1	Haloacid dehalogenase-like hydrolase (HAD) superfamily protein//Phosphorylated carbohydrates phosphatase TM_1254	1142.463
Ciclev10032556m|PACid:20804067	AT2G38740.1	Haloacid dehalogenase-like hydrolase domain-containing protein Sgpp	3.182
Ciclev10016366m|PACid:20815584	AT5G44730.1	Haloacid dehalogenase-like hydrolase (HAD) superfamily protein	309.403
Ciclev10008400m|PACid:20795268	AT2G25870.1	Haloacid dehalogenase-like hydrolase family protein	0.415
**Nucleic acid metabolism**			
Ciclev10009937m|PACid:20796049	AT5G26170.1	Probable WRKY transcription factor 50	3.042
Ciclev10009720m|PACid:20795220	AT5G11260.1	Basic-leucine zipper (bZIP) transcription factor family protein//Protein LONG HYPOCOTYL 5//Transcription factor HY5//bZIP transcription factor 56	17.626
Ciclev10002081m|PACid:20786258	AT3G12480.1	Nuclear factor Y, subunit C11	23.136
Ciclev10025390m|PACid:20800887	AT5G66730.1	C2H2-like zinc finger protein	70.035
Ciclev10033000m|PACid:20804271		HAP3-like protein	2.050
Ciclev10016303m|PACid:20816503	AT3G23250.1	Myb domain protein 15	0.310
Ciclev10004185m|PACid:20791644	AT5G49930.1	Zinc knuckle (CCHC-type) family protein	0.438
Ciclev10015532m|PACid:20815484	AT5G07900.1	Mitochondrial transcription termination factor family protein	0.459
Ciclev10010753m|PACid:20792790	AT1G15340.1	Methyl-CPG-binding domain 11	2.060
Ciclev10033914m|PACid:20802816	AT3G12640.1	RNA-binding (RRM/RBD/RNP motifs) family protein	368.818
Ciclev10025862m|PACid:20801963	AT2G18510.1	RNA-binding (RRM/RBD/RNP motifs) family protein//Splicing factor 3B subunit 4	293.896
Ciclev10014272m|PACid:20817277	AT3G02010.1	Pentatricopeptide repeat (PPR) superfamily protein	611.788
Ciclev10018370m|PACid:20818993	AT4G03430.1	Putative pre-mRNA splicing factor	2.190
Ciclev10030532m|PACid:20804921	AT3G55220.1	Spliceosome-associated protein 130	0.496
Ciclev10021761m|PACid:20808316	AT1G18800.1	NAP1-related protein 2	0.493
Ciclev10004916m|PACid:20791356	AT3G54470.1	Uridine 5′-monophosphate synthase	543.655
Ciclev10007580m|PACid:20793771	AT5G11350.1	DNase I-like superfamily protein	0.466
**Protein and amino acid metabolism**			
Ciclev10004211m|PACid:20790583	AT5G49030.3	tRNA synthetase class I (I, L, M and V) family protein	0.417
Ciclev10004807m|PACid:20791740	AT3G06430.1	Tetratricopeptide repeat (TPR)-like superfamily protein	0.322
Ciclev10025061m|PACid:20800270	AT5G08650.1	Elongation factor 4 homolog/Ribosomal back-translocase/Small GTP-binding protein/Translation factor GUF1 homolog, chloroplastic	0.489
Ciclev10006396m|PACid:20789804	ATCG00800.1	Structural constituent of ribosome	0.453
Ciclev10022283m|PACid:20810800	AT3G49010.1	60S ribosomal protein L13/Protein BBC1 homolog/Breast basic conserved 1	0.361
Ciclev10013047m|PACid:20799080	AT3G56910.1	Plastid-specific 50S ribosomal protein 5	0.348
Ciclev10013049m|PACid:20798310	AT2G38140.1	Plastid-specific ribosomal protein 4	0.319
Ciclev10009636m|PACid:20793290	AT3G15190.1	Chloroplast 30S ribosomal protein S20, putative	0.494
Ciclev10022870m|PACid:20811532	AT3G05560.1	Ribosomal L22e protein family	0.465
Ciclev10006136m|PACid:20789692	AT2G33450.1	Ribosomal L28 family	0.298
Ciclev10009798m|PACid:20796123	AT2G19730.1	Ribosomal L28e protein family	0.470
Ciclev10026623m|PACid:20801707	AT5G65220.1	Ribosomal L29 family protein	0.384
Ciclev10033083m|PACid:20804008	AT2G39390.1	Ribosomal L29 family protein	0.169
Ciclev10016875m|PACid:20816724	AT5G45775.2	Ribosomal L5P family protein	0.473

Table S1. *Cont.*

Accession	Homology in *Arabidopsis thaliana*	Proteins	Fold Change (121/119)
**Protein and amino acid metabolism**			
Ciclev10021696m|PACid:20808755	AT4G01310.1	Ribosomal L5P family protein	0.439
Ciclev10032705m|PACid:20803904	AT5G13510.1	Ribosomal protein L10 family protein	0.446
Ciclev10021899m|PACid:20808624	AT1G78630.1	Ribosomal protein L13 family protein	0.396
Ciclev10023476m|PACid:20812085	ATCG00780.1	Ribosomal protein L14	0.448
Ciclev10016274m|PACid:20815504	AT3G25920.1	Ribosomal protein L15	0.451
Ciclev10004001m|PACid:20788151	ATCG00790.1	Ribosomal protein L16	0.429
Ciclev10032703m|PACid:20802569	AT1G26910.1	Ribosomal protein L16p/L10e family protein	0.397
Ciclev10006182m|PACid:20792369	AT1G02780.1	Ribosomal protein L19e family protein	0.355
Ciclev10030134m|PACid:20813406	AT5G22440.1	Ribosomal protein L1p/L10e family	0.487
Ciclev10016308m|PACid:20817308	AT4G36130.1	Ribosomal protein L2 family	0.273
Ciclev10002401m|PACid:20788696	AT1G35680.1	Ribosomal protein L21	0.353
Ciclev10005921m|PACid:20792152	ATCG00810.1	Ribosomal protein L22	0.393
Ciclev10023969m|PACid:20810966	AT3G53020.1	Ribosomal protein L24	0.393
Ciclev10017015m|PACid:20815733	AT1G29070.1	Ribosomal protein L34	0.343
Ciclev10022900m|PACid:20806865	AT1G26880.1	Ribosomal protein L34e superfamily protein	0.302
Ciclev10033093m|PACid:20804391	AT1G26880.1	Ribosomal protein L34e superfamily protein	0.295
Ciclev10013164m|PACid:20798847	AT1G07070.1	Ribosomal protein L35Ae family protein	0.399
Ciclev10006273m|PACid:20790606	AT3G53740.2	Ribosomal protein L36e family protein	0.382
Ciclev10011875m|PACid:20799017	AT3G09630.1	Ribosomal protein L4/L1 family	0.456
Ciclev10020582m|PACid:20810676	AT3G25520.1	Ribosomal protein L5	0.388
Ciclev10016107m|PACid:20818376	AT5G39740.1	Ribosomal protein L5 B	0.412
Ciclev10009377m|PACid:20794151	AT1G33140.1	Ribosomal protein L6 family	0.483
Ciclev10016578m|PACid:20816737	AT1G05190.1	Ribosomal protein L6 family	0.459
Ciclev10032636m|PACid:20805408	AT1G74050.1	Ribosomal protein L6 family protein	0.463
Ciclev10032494m|PACid:20803323	AT2G47610.1	Ribosomal protein L7Ae/L30e/S12e/Gadd45 family protein	0.433
Ciclev10006259m|PACid:20792027	AT1G77940.1	Ribosomal protein L7Ae/L30e/S12e/Gadd45 family protein	0.346
Ciclev10029219m|PACid:20813782	AT3G44890.1	Ribosomal protein L9	0.440
Ciclev10016825m|PACid:20818990	AT5G40950.1	Ribosomal protein large subunit 27	0.458
Ciclev10009763m|PACid:20793057	AT5G23740.1	Ribosomal protein S11-beta	0.396
Ciclev10002692m|PACid:20789374	AT5G14320.1	Ribosomal protein S13/S18 family	0.387
Ciclev10006145m|PACid:20790323	AT4G00100.1	Ribosomal protein S13A	0.413
Ciclev10026679m|PACid:20800350	AT1G79850.1	Ribosomal protein S17	0.475
Ciclev10003202m|PACid:20788933	ATCG00650.1	Ribosomal protein S18	0.463
Ciclev10002819m|PACid:20786600	AT3G04920.1	Ribosomal protein S24e family protein	0.402
Ciclev10029630m|PACid:20813886	AT2G21580.2	Ribosomal protein S25 family protein	0.486
Ciclev10002825m|PACid:20785887	AT3G56340.1	Ribosomal protein S26e family protein	0.464
Ciclev10009868m|PACid:20794701	AT5G56670.1	Ribosomal protein S30 family protein	0.208
Ciclev10012508m|PACid:20798906	AT4G34670.1	Ribosomal protein S3Ae	0.445

Table S1. *Cont.*

Accession	Homology in *Arabidopsis thaliana*	Proteins	Fold Change (121/119)
**Protein and amino acid metabolism**			
Ciclev10032800m|PACid:20804334	AT5G39850.1	Ribosomal protein S4	0.444
Ciclev10029508m|PACid:20812895	AT2G09990.1	Ribosomal protein S5 domain 2-like superfamily protein	0.497
Ciclev10006162m|PACid:20790988	AT2G09990.1	Ribosomal protein S5 domain 2-like superfamily protein	0.382
Ciclev10005613m|PACid:20790205	AT3G57490.1	Ribosomal protein S5 family protein	0.452
Ciclev10026340m|PACid:20801998	AT5G10360.1	Ribosomal protein S6e	0.464
Ciclev10012856m|PACid:20796870	AT5G16130.1	Ribosomal protein S7e family protein	0.478
Ciclev10009415m|PACid:20792807	AT5G59240.1	Ribosomal protein S8e family protein	0.370
Ciclev10023063m|PACid:20807474	AT3G10950.1	Zinc-binding ribosomal protein family protein	0.458
Ciclev10022990m|PACid:20811127	AT4G14320.1	Zinc-binding ribosomal protein family protein	0.441
Ciclev10026841m|PACid:20799774	AT3G16080.1	Ribosomal protein L37//Zinc-binding ribosomal protein family protein	0.235
Ciclev10009628m|PACid:20794614	AT5G54600.1	Translation protein SH3-like family protein	0.492
Ciclev10026719m|PACid:20802119	AT3G49910.1	Translation protein SH3-like family protein	0.428
Ciclev10012788m|PACid:20797116	AT1G57860.1	Translation protein SH3-like family protein	0.347
Ciclev10001454m|PACid:20789331	AT1G43170.8	60S ribosomal protein L3//ribosomal protein 1	0.424
Ciclev10001453m|PACid:20788226	AT1G61580.1	60S ribosomal protein L3-2//R-protein L3 B	2.068
Ciclev10005170m|PACid:20789963	AT4G13670.1	Plastid transcriptionally active 5	2.043
Ciclev10012870m|PACid:20798639	AT3G12390.1	Nascent polypeptide-associated complex (NAC), alpha subunit family protein	374.508
Ciclev10022215m|PACid:20807094	AT3G49470.1	Nascent polypeptide-associated complex subunit alpha-like protein 2	73.723
Ciclev10026634m|PACid:20801099	AT4G37660.1	50S ribosomal protein L7/L12//Ribosomal protein L12/ ATP-dependent Clp protease adaptor protein ClpS family protein	196.542
Ciclev10018845m|PACid:20809345	AT1G56070.1	Ribosomal protein S5/Elongation factor G/III/V family protein	576.342
Ciclev10029555m|PACid:20813972	AT5G02960.1	Ribosomal protein S12/S23 family protein	25.756
Ciclev10009899m|PACid:20793086	AT5G20160.1	Ribosomal protein L7Ae/L30e/S12e/Gadd45 family protein	22.210
Ciclev10022435m|PACid:20810058	AT5G27850.1	Ribosomal protein L18e/L15 superfamily protein	71.594
Ciclev10003535m|PACid:20787040	AT1G35340.1	ATP-dependent protease La (LON) domain protein	2.960
Ciclev10015814m|PACid:20818497	AT2G27420.1	Cysteine proteinases	315.813
Ciclev10020859m|PACid:20807278	AT1G02305.1	Cysteine proteinases superfamily protein	2.147
Ciclev10005038m|PACid:20790402	AT1G03220.1	Eukaryotic aspartyl protease family protein	2.260
Ciclev10001060m|PACid:20785798	AT1G73300.1	Serine carboxypeptidase, putative//Serine carboxypeptidase-like 18	2.432
Ciclev10029139m|PACid:20813954	AT5G66140.1	Proteasome α subunit D2	1116.926
Ciclev10026339m|PACid:20801160	AT5G66140.1	Proteasome α subunit D2	975.112
Ciclev10014351m|PACid:20817403	AT2G04160.1	Subtilisin-like serine endopeptidase family protein	310.100
Ciclev10001054m|PACid:20785799	AT1G47128.1	Granulin repeat cysteine protease family protein	2.617
Ciclev10002346m|PACid:20788131	AT3G12490.2	Cystatin B	2.256
Ciclev10003705m|PACid:20786256	AT5G47550.1	Cystatin/monellin superfamily protein	2.037
Ciclev10022001m|PACid:20806490	AT1G17860.1	Kunitz family trypsin and protease inhibitor protein	3.039
Ciclev10022156m|PACid:20808320	AT1G17860.1	Kunitz family trypsin and protease inhibitor protein	2.537
Ciclev10022211m|PACid:20810798	AT1G17860.1	Kunitz family trypsin and protease inhibitor protein	2.293
Ciclev10011794m|PACid:20796925	AT2G24280.1	α/β-Hydrolases superfamily protein	2.007

Table S1. *Cont.*

Accession	Homology in *Arabidopsis thaliana*	Proteins	Fold Change (121/119)
**Protein and amino acid metabolism**			
Ciclev10021018m|PACid:20812099	AT4G10790.1	UBX domain-containing protein	346.152
Ciclev10023537m|PACid:20809334	AT4G17510.1	Ubiquitin C-terminal hydrolase 3	597.747
Ciclev10009208m|PACid:20795336	AT5G25770.3	α/β-Hydrolases superfamily protein	0.283
Ciclev10022124m|PACid:20806488	AT1G17860.1	Kunitz family trypsin and protease inhibitor protein	0.454
Ciclev10008097m|PACid:20793654	AT4G32940.1	Gamma vacuolar processing enzyme	0.454
Ciclev10014528m|PACid:20815760	AT5G46210.1	Cullin4	0.498
Ciclev10032969m|PACid:20804504	AT2G47110.1	Ubiquitin 6	0.268
Ciclev10008698m|PACid:20793182	AT5G05920.1	Deoxyhypusine synthase	110.347
Ciclev10028599m|PACid:20813645	AT5G64370.1	Beta-alanine synthase//*N*-carbamoyl-beta-alanine amidohydrolase//Predicted protein	294.686
Ciclev10021002m|PACid:20809681	AT4G08900.1	Arginase	2.541
Ciclev10011621m|PACid:20797657	AT3G08860.1	Alanine-glyoxylate aminotransferase 2 homolog 3, mitochondrial//Alanine-glyoxylate aminotransferase, putative	2.110
Ciclev10019032m|PACid:20806055	AT4G12290.1	Copper amine oxidase family protein	2.846
Ciclev10015485m|PACid:20818727	AT2G13360.1	Alanine:glyoxylate aminotransferase	0.382
Ciclev10007310m|PACid:20794460	AT4G33010.1	Glycine decarboxylase P-protein 1	0.393
Ciclev10033729m|PACid:20804854	AT1G69800.2	Cystathionine β-synthase (CBS) protein	0.423
Ciclev10032361m|PACid:20805672	AT1G07780.1	Phosphoribosylanthranilate isomerase 1	0.227
Ciclev10027913m|PACid:20814546	AT2G37040.1	Phenylalanine ammonia-lyase 1	0.325
Ciclev10001828m|PACid:20788451	AT1G76730.1	Methenyltetrahydrofolate synthase domain-containing protein//NagB/RpiA/CoA transferase-like superfamily protein	0.486
**Cell wall and cytoskeleton metabolism**			
Ciclev10021884m|PACid:20807416	AT4G17030.1	Expansin-like B1	3.008
Ciclev10033057m|PACid:20802906	AT5G59890.2	Actin-depolymerizing factor 2//Actin depolymerizing factor 4	2188.468
Ciclev10011723m|PACid:20796428	AT5G23860.2	Beta-8-tubulin	15.113
Ciclev10011719m|PACid:20798687	AT5G12250.1	Tubulin beta-6 chain	0.383
Ciclev10028426m|PACid:20814187	AT1G50010.1	Tubulin α-2 chain	0.433
**Cellular transport**			
Ciclev10013168m|PACid:20798707	AT3G01390.1	V-type proton ATPase subunit G//Vacuolar proton pump subunit G//Vacuolar membrane ATPase 10	540.092
Ciclev10032471m|PACid:20804478	AT2G40300.1	Ferritin 4	2.200
Ciclev10029240m|PACid:20812600	AT2G16060.1	Non-symbiotic hemoglobin 1	2.113
Ciclev10006560m|PACid:20791490	AT5G51720.1	CDGSH iron-sulfur domain-containing protein NEET	2.146
Ciclev10012107m|PACid:20798800	AT2G40060.1	Clathrin light chain 2//Clathrin light chain protein	2.006
Ciclev10016438m|PACid:20816862	AT1G61250.1	Secretory carrier-associated membrane protein 3//secretory carrier 3	9.400
Ciclev10032366m|PACid:20805747	AT3G01280.1	Voltage dependent anion channel 1	3.454
Ciclev10006198m|PACid:20791190	AT1G01490.2	Heavy-metal-associated domain-containing protein	2.917
Ciclev10020395m|PACid:20809498	AT2G36070.1	Mitochondrial import inner membrane translocase subunit TIM44-2	2.032
Ciclev10008825m|PACid:20793014	AT4G30840.1	Nucleoporin Nup43//Nup107-160 subcomplex subunit Nup43//Transducin/WD40 repeat-like superfamily protein	166.926
Ciclev10007941m|PACid:20794860	AT2G18710.1	Preprotein translocase subunit SCY1, chloroplastic	0.241
Ciclev10014606m|PACid:20818166	AT1G14830.1	Dynamin	0.484
Ciclev10000332m|PACid:20789395	AT1G76850.1	Exocyst complex component 2//Exocyst complex component Sec5//Predicted protein	0.412

Table S1. *Cont.*

Accession	Homology in *Arabidopsis thaliana*	Proteins	Fold Change (121/119)
**Cellular transport**			
Ciclev10014840m|PACid:20817945	AT4G25650.1	ACD1-like	0.464
Ciclev10007246m|PACid:20793324	AT3G43300.1	HOPM interactor 7	0.449
Ciclev10030708m|PACid:20805624	AT2G35800.1	Mitochondrial substrate carrier family protein	0.470
Ciclev10006055m|PACid:20792243	AT4G14870.1	Preprotein translocase subunit SECE1//SecE/SEC61-gamma protein transport protein	0.363
Ciclev10018709m|PACid:20805894	AT2G46520.1	Cellular apoptosis susceptibility protein homolog//Exportin-2//Importin-alpha re-exporter	0.498
Ciclev10007348m|PACid:20795660	AT5G11560.1	Catalytic, putative//ER membrane protein complex subunit 1//Catalytics	0.498
**Lipid metabolism**			
Ciclev10032510m|PACid:20802930	AT3G25780.1	Allene oxide cyclase 3	111.562
Ciclev10014204m|PACid:20816226	AT3G45140.1	Lipoxygenase 2	3.025
Ciclev10004649m|PACid:20791288	AT3G16910.1	Acetate/butyrate-CoA ligase AAE7, peroxisomal//Acyl-activating enzyme 7	195.305
Ciclev10022587m|PACid:20810567	AT3G61200.1	Acyl-CoA thioesterase, putative//Thioesterase superfamily protein	150.409
Ciclev10020242m|PACid:20811380	AT1G01710.1	Acyl-CoA thioesterase family protein	4.523
Ciclev10019200m|PACid:20807021	AT4G23850.1	Long chain acyl-CoA synthetase 4	0.449
Ciclev10010427m|PACid:20796029	AT1G53920.1	GDSL esterase/lipase 5	2.526
Ciclev10020550m|PACid:20810248	AT3G60510.1	3-hydroxyisobutyryl-CoA hydrolase-like protein 1, mitochondrial	61.285
Ciclev10031489m|PACid:20804761	AT5G17540.1	HXXXD-type acyl-transferase family protein	2.148
Ciclev10025939m|PACid:20800257	AT4G39330.1	Cinnamyl alcohol dehydrogenase 9	0.475
Ciclev10020721m|PACid:20807101	AT4G00400.1	Glycerol-3-phosphate acyltransferase 8	0.371
Ciclev10026558m|PACid:20800793	AT5G48930.1	Hydroxycinnamoyl transferase	0.365
Ciclev10013542m|PACid:20799197	AT5G54160.1	O-methyltransferase 1	0.383
Ciclev10008761m|PACid:20795154	AT4G33110.1	Coclaurine *N*-methyltransferase	0.417
Ciclev10010749m|PACid:20792814	AT3G18000.1	Phosphoethanolamine *N*-methyltransferase 1//Phosphoethanolamine *N*-methyltransferase, putative	0.232
Ciclev10033579m|PACid:20803972	AT5G19290.1	HU-K5//Lysophospholipase-like//Monoacylglycerol lipase//Monoglyceride lipase	0.488
**Signal transduction**			
Ciclev10031755m|PACid:20803828	AT1G14000.1	VH1-interacting kinase	2.151
Ciclev10012334m|PACid:20797162	AT5G39790.1	5′-AMP-activated protein kinase-related	2.088
Ciclev10006977m|PACid:20789643	AT5G52200.1	Phosphoprotein phosphatase inhibitors	1700.715
Ciclev10002098m|PACid:20789158	AT1G34750.1	Protein phosphatase 2C family protein	22.049
Ciclev10021603m|PACid:20811358	AT4G11240.1	Serine/threonine-protein phosphatase PP1 isozyme 6	2.246
Ciclev10009194m|PACid:20796151	AT5G06740.1	Concanavalin A-like lectin protein kinase family protein	0.250
Ciclev10018681m|PACid:20806123	AT5G23060.1	Calcium sensing receptor	360.223
Ciclev10017002m|PACid:20819306	AT5G47710.1	Calcium-dependent lipid-binding (CaLB domain) family protein	52.792
Ciclev10005548m|PACid:20792350	AT3G16510.1	Calcium-dependent lipid-binding (CaLB domain) family protein	18.237
Ciclev10005575m|PACid:20789955	AT3G10300.3	Calcium-binding EF-hand family protein//Calmodulin-like protein 49//Probable calcium-binding protein CML49	7.611
Ciclev10003554m|PACid:20787567	AT2G21830.1	Cysteine/Histidine-rich C1 domain family protein	2.480
Ciclev10032615m|PACid:20804275	AT3G07390.1	Auxin-induced in root cultures protein 12//Auxin-responsive family protein	456.371
Ciclev10022378m|PACid:20809005	AT4G01900.1	GLNB1 homolog//Nitrogen regulatory protein P-II homolog//Protein PII-like	3.131
Ciclev10000962m|PACid:20788421	AT5G63910.1	Farnesylcysteine lyase	143.231
Ciclev10021008m|PACid:20809776	AT3G59380.1	Farnesyltransferase A	0.374

Table S1. *Cont.*

Accession	Homology in *Arabidopsis thaliana*	Proteins	Fold Change (121/119)
**Others**			
Ciclev10002486m|PACid:20787718	AT4G31950.1	Cytochrome P450	0.226
Ciclev10007096m|PACid:20791221	AT1G64940.1	Cytochrome P450, family 87, subfamily A, polypeptide 6	0.467
Ciclev10004785m|PACid:20790316	AT3G26300.1	Cytochrome P450, family 71, subfamily B, polypeptide 34	0.239
Ciclev10000866m|PACid:20786710	AT3G26300.1	Cytochrome P450, family 71, subfamily B, polypeptide 34	0.403
Ciclev10031280m|PACid:20805703	AT3G26300.1	Cytochrome P450, family 71, subfamily B, polypeptide 34	0.299
Ciclev10017346m|PACid:20815057	AT5G04750.1	F1F0-ATPase inhibitor protein, putative	2.385
Ciclev10005457m|PACid:20791540	AT5G24020.1	Septum site-determining protein (MIND)	9.392
Ciclev10021578m|PACid:20810267	AT1G53520.1	Chalcone-flavonone isomerase 2	17.473
Ciclev10001946m|PACid:20787842	AT1G75280.1	Isoflavone reductase homolog P3	0.401
Ciclev10008386m|PACid:20794913	AT5G53860.2	Embryo defective 2737	12.485
Ciclev10012793m|PACid:20797650	AT2G04690.1	Pyridoxamine 5′-phosphate oxidase family protein	2.225
Ciclev10031003m|PACid:20805689	AT4G19170.1	Probable carotenoid cleavage dioxygenase 4, chloroplastic	0.315
Ciclev10000425m|PACid:20787673	AT1G21680.1	DPP6 N-terminal domain-like protein	4.505
Ciclev10005724m|PACid:20790262	AT3G23600.1	α/β-Hydrolases superfamily protein	2.136
Ciclev10015803m|PACid:20818801	AT2G36290.1	α/β-Hydrolases superfamily protein	0.405
Ciclev10014035m|PACid:20817344	AT5G47690.3	Uncharacterized binding protein	0.427
Ciclev10016821m|PACid:20815954	AT5G14030.1	Translocon-associated protein beta (TRAPB) family protein	15.251
Ciclev10026171m|PACid:20801938	AT4G36850.1	PQ-loop repeat family protein/transmembrane family protein	2.084
Ciclev10016230m|PACid:20818084	AT4G21470.1	Bifunctional riboflavin kinase/FMN phosphatase	2.259
Ciclev10008512m|PACid:20793764	AT1G04970.1	Lipid-binding serum glycoprotein family protein	2.080
Ciclev10001991m|PACid:20787158	AT3G04830.1	Protein prenylyltransferase superfamily protein	0.473
Ciclev10020109m|PACid:20808858	AT5G50150.1	Protein of Unknown Function (DUF239)	2.983
Ciclev10026423m|PACid:20799876	AT1G15860.1	DCN1-like protein 4//DCUN1 domain-containing protein 4//Defective in cullin neddylation protein 1-like protein 4	0.499
Ciclev10016630m|PACid:20817048	AT3G20790.1	NAD(P)-binding Rossmann-fold superfamily protein	0.393

Table S2. List of differentially expressed proteins in Al-toxic *Citrus grandis* leaves. Control and Al-toxic samples were labeled with 114 and 117 tags, respectively.

Accession	Homology in *Arabidopsis thaliana*	Proteins	Fold Change (117/114)
**Energy and carbohydrate metabolism**			
Ciclev10016116m|PACid:20816432	AT3G26710.1	Cofactor assembly of complex C	2.072
**S metabolism**			
Ciclev10002119m|PACid:20786689	AT2G47730.1	Glutathione *S*-transferase ϕ 8	0.431
**Stress response**			
Ciclev10009502m|PACid:20793097	AT2G24440.1	Selenium binding	0.457
**Low-P-responsive proteins**			
Ciclev10026096m|PACid:20800141	AT1G73010.1	Inorganic pyrophosphatase 1//Predicted protein	2.808
Ciclev10015568m|PACid:20815289	AT3G02040.1	Glycerophosphodiester phosphodiesterase GDE1	2.407
**Nucleic acid metabolism**			
Ciclev10014026m|PACid:20818243	AT3G01460.1	Methyl-CPG-binding domain 9	4.615
Ciclev10031009m|PACid:20805801	AT3G48710.1	DEK domain-containing chromatin associated protein	0.422
**Protein and amino acid metabolism**			
Ciclev10004119m|PACid:20790317	AT1G65810.1	P-loop containing nucleoside triphosphate hydrolases superfamily protein	0.441
Ciclev10029619m|PACid:20813697	AT3G44590.1	60S acidic ribosomal protein family	0.412
Ciclev10007222m|PACid:20793486	AT1G55860.2	Ubiquitin-protein ligase 1	2.416
**Cellular transport**			
Ciclev10025542m|PACid:20801125	AT1G80310.1	Molybdate transporter 2//Sulfate transporter like protein 5.1//Sulfate transporter, putative//Sulfate transmembrane transporters	2.623
Ciclev10005024m|PACid:20791376	AT1G05370.1	Sec14p-like phosphatidylinositol transfer family protein	0.282
**Signal transduction**			
Ciclev10027740m|PACid:20813291	AT3G45780.1	Non-phototropic hypocotyl protein 1//Root phototropism protein 1	0.456
Ciclev10009194m|PACid:20796151	AT5G06740.1	Concanavalin A-like lectin protein kinase family protein	0.439
**Others**			
Ciclev10008649m|PACid:20793993	AT4G27520.1	Early nodulin-like protein 2	0.460
Ciclev10009420m|PACid:20795288	AT3G20570.1	Early nodulin-like protein 9	0.325
Ciclev10032528m|PACid:20805031	AT3G55605.1	Mitochondrial glycoprotein family protein	0.468

Table S3. Specific primer pairs used for qRT-PCR expression analysis.

Accession	Proteins	Forward Primers (5′→3′)	Reverse Primers (5′→3′)
Ciclev10015568m|PACid:20815289	Glycerophosphodiester phosphodiesterase GDE1	AATCGGCGTCGTTTAG	GGGATATTTAGCGGAAGA
Ciclev10025542m|PACid:20801125	Molybdate transporter 2	CTCCTCGCCCTCACAG	GGGTATTGCCGACAGA
Ciclev10002119m|PACid:20786689	Glutathione *S*-transferase phi 8	TGGCAACTCCAGTAAAA	ACAAACATAGCGGCATA
Ciclev10009420m|PACid:20795288	Early nodulin-like protein 9	GCCAGCAAATGGAGTA	CACGAGGATGAGGATAAT
Ciclev10008649m|PACid:20793993	Early nodulin-like protein 2	AACTGGATTCGGGTGA	GGAGACTTAGGAGACGG
Ciclev10004927m|PACid:20791536	ATP sulfurylase 1	TGCTCCTCCCCTCACA	ACTTCCAAAGCCAATGAGACT
Ciclev10000272m|PACid:20786660	1,4-α-glucan-branching enzyme//Q-enzyme	GGATGGAGGACGAGACAG	GACAACAATAAGGAATACGAGGA
Ciclev10019346m|PACid:20806605	Granule-bound starch synthase 1, chloroplastic	GGAACCCCTTGACTGA	CGGACGAATGAATGTGGGT
Ciclev10018845m|PACid:20809345	Ribosomal protein S5/Elongation factor G/III/V family protein	GCTGCCCTTCGTATCACT	TCTCCTCACTCAATCCCAACT
Ciclev10008097m|PACid:20793654	Gamma vacuolar processing enzyme	GCTCCAGCAACCAATC	GGTTTATGTTGGAGGAGTAGGAT
Ciclev10001726m|PACid:20787677	ATP4a//PRXR6//Peroxidase 12	CCCAGTAGCGAAAGGT	GAGGGACTAAAGGACCGACA
Ciclev10022164m|PACid:20810119	Glutathione *S*-transferase τ 19	TTACTTTCGGCGTGTT	ACCAGAAGCCAGCAACAAT
Ciclev10028656m|PACid:20812272	(1->3)-β-glucan endohydrolase 7	CATCCGCCAAGACACTAT	CCGCACAAATGATTCCAGT
Ciclev10000951m|PACid:20786358	Catalase	TTTTCTGCTTCCCTCC	CAAGAAGCCCAAGGAAGC
Ciclev10007941m|PACid:20794860	Preprotein translocase subunit SCY1	TCTCCCAGACTCAGAAAC	TGCAAGTGGCTGAACAGAA
Ciclev10010749m|PACid:20792814	Phosphoethanolamine *N*-methyltransferase 1	TGGCAGAAAGGATGGT	AGGGCTATAATGGGCAGTG
Ciclev10006055m|PACid:20792243	Preprotein translocase subunit SECE1	AAAGCCGCCACCTGTA	CAGATAGCCAAGAAGAGGAGC
Ciclev10015870m|PACid:20817415	ATP1a/ATP1b//PRXR1	CCTTTAGTGAGTGAAGAGTAG	GGACTACCGTAATAGTAAAGTCGC
Ciclev10027913m|PACid:20814546	Phenylalanine ammonia-lyase 1	ACGGAACAGGAGTGGG	CCGTAACCATTCCCTCCATC
Ciclev10022212m|PACid:20809459	Glutathione S-transferase F8, chloroplastic	CACGGTTCTGTTTTCTCA	TTGGGGAGCAGGTTGGA
GU911361.1	Actin	AGAACTATGAACTGCCTGATGGC	GCTTGGAGCAAGTGCTGTGATT

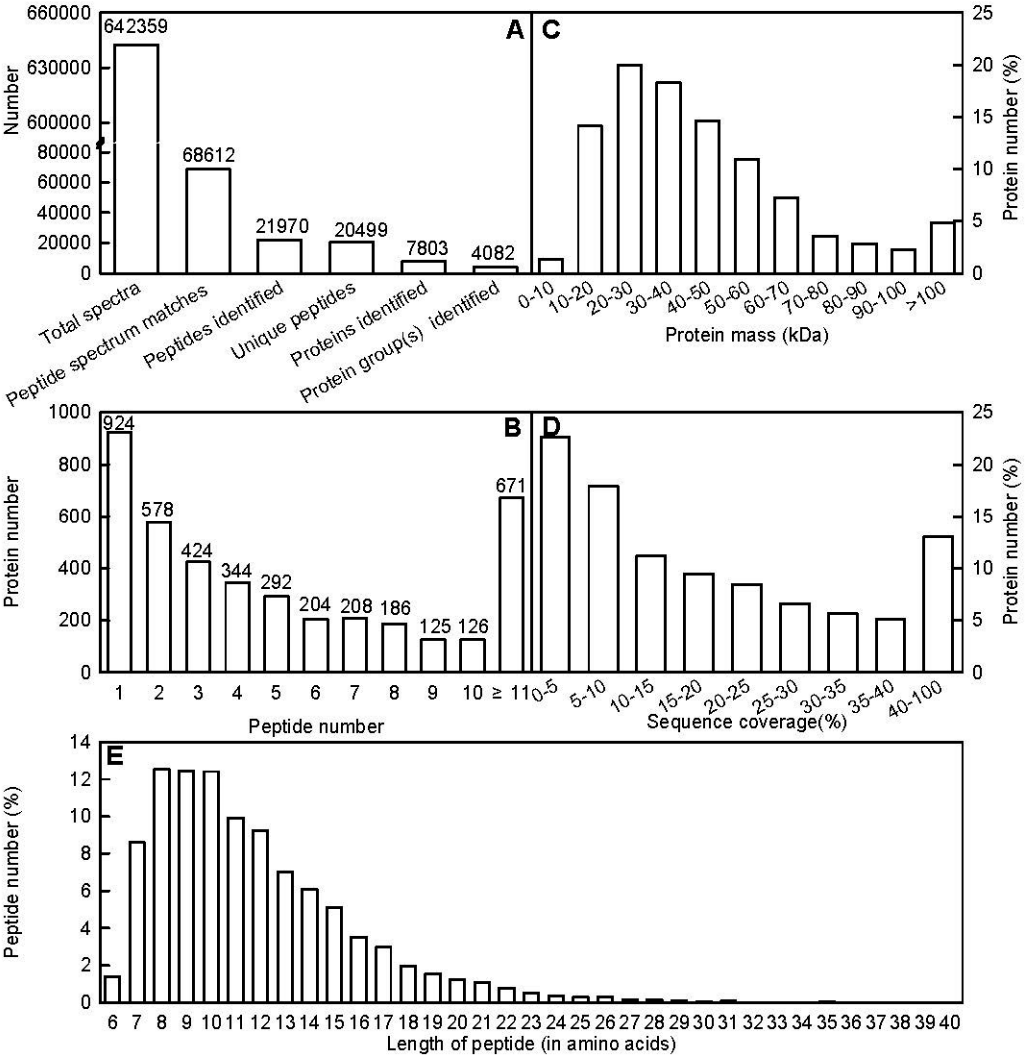

Figure S1. Spectra, peptides and proteins identified from iTRAQ proteomics (**A**); number of peptides that match to proteins (**B**); protein mass distribution (**C**); distribution of protein sequence coverage (**D**); and distribution of peptide length (**E**) in *C. sinensis* leaves.

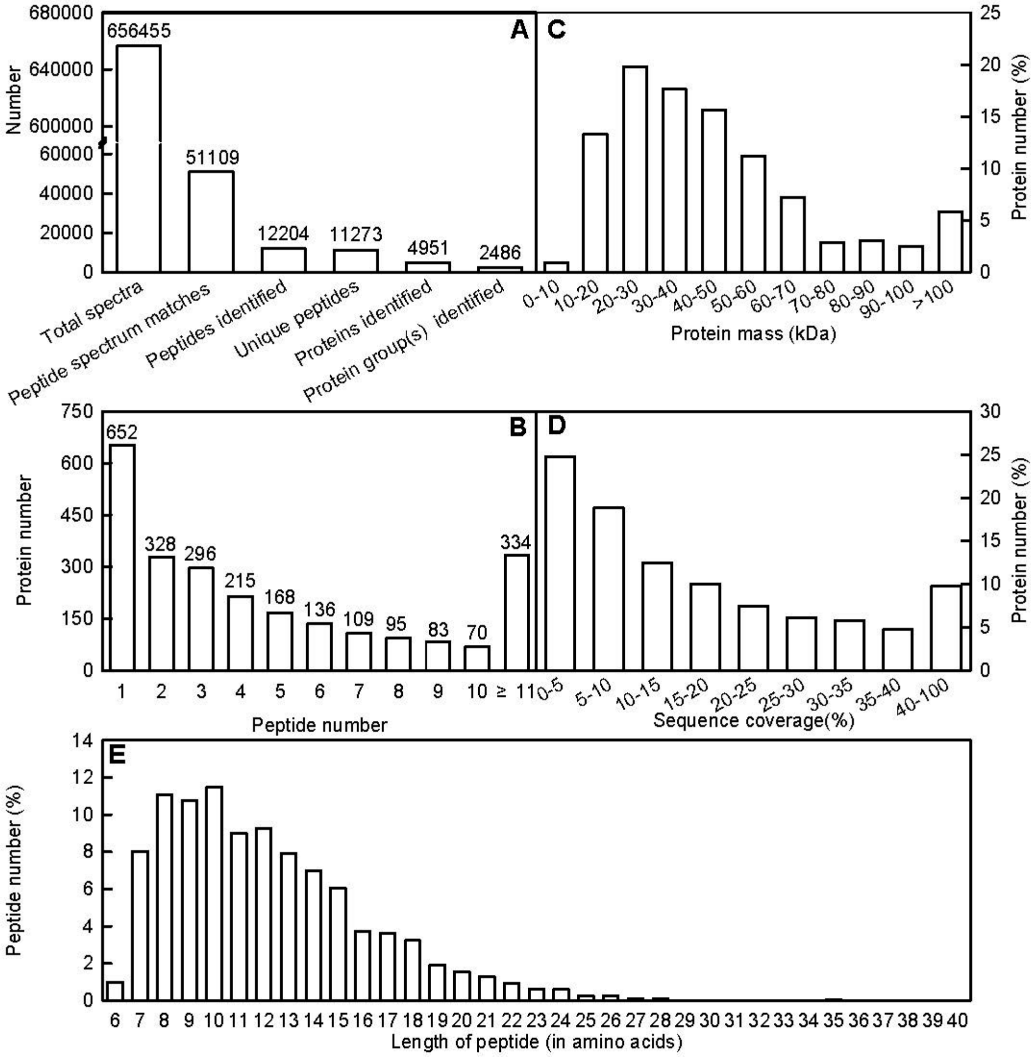

Figure S2. Spectra, peptides and proteins identified from iTRAQ proteomics (**A**); number of peptides that match to proteins (**B**); protein mass distribution (**C**); distribution of protein sequence coverage (**D**); and distribution of peptide length (**E**) in *C. grandis* leaves.

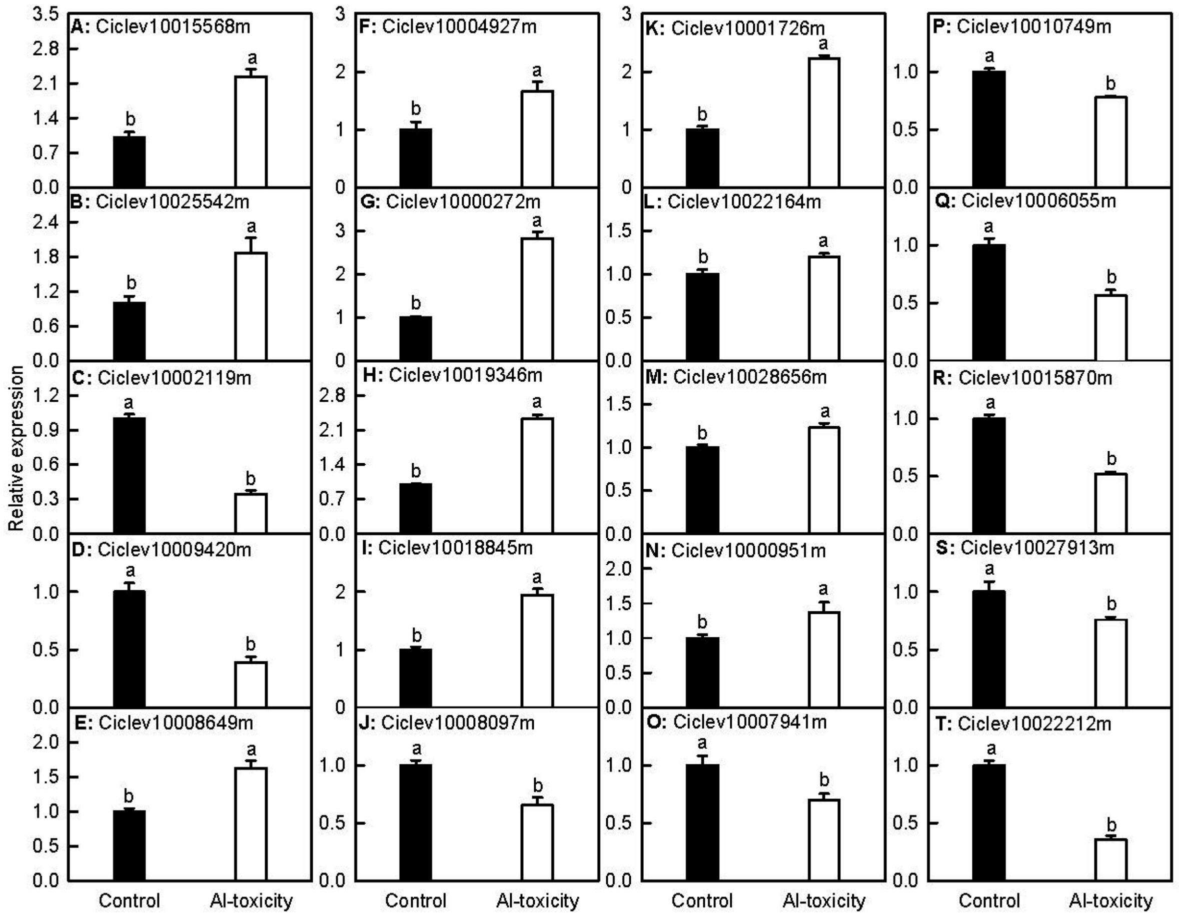

Figure S3. Relative expression levels of genes for differentially abundant proteins from *C. grandis* (**A**–**E**) and *C. sinensis* (**F**–**T**) leaves. qRT-PCR was run in three biological replicates with two technical replicates. For the normalization of gene, citrus *actin* (GU911361.1) was used as an internal standard and the sample from control plants was used as reference sample, which was set to 1. Bars represent means ± standard error. Significant tests between means were carried out by unpaired *t*-test. Different letters above the bars indicate a significant difference at $p < 0.05$.

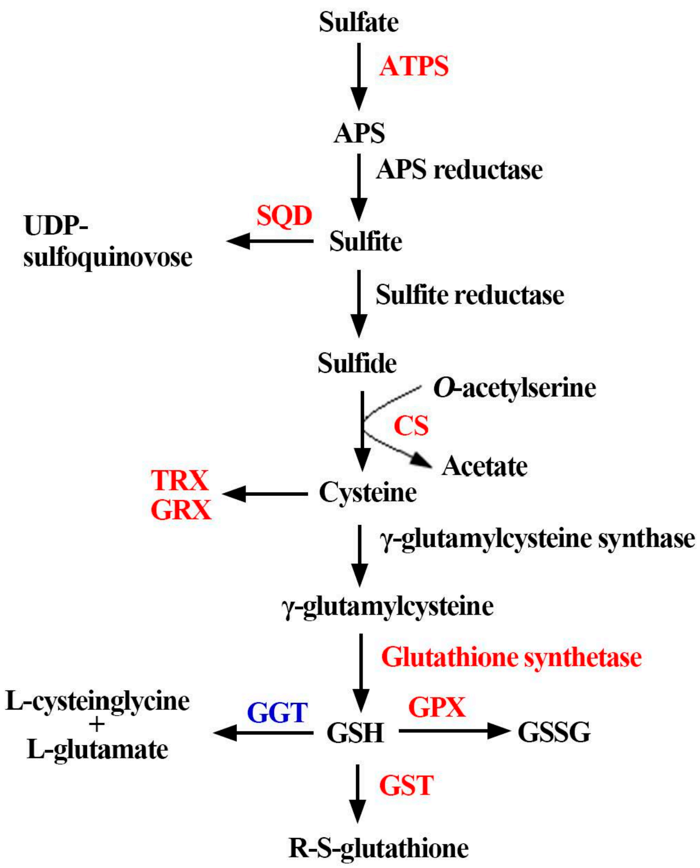

Figure S4. Al-induced alterations of S metabolism in *C. sinensis* leaves. Proteins with increased (decreased) abundances were highlighted in red (blue). APS: adenosine phosphosulphate; GGT: gamma-glutamyl transpeptidase; GST: glutathione *S*-transferase; SQD: UDP-sulfoquinovose synthase.
